# Advances in 3D Bioprinting: Materials, Processes, and Emerging Applications

**DOI:** 10.3390/mi17030282

**Published:** 2026-02-25

**Authors:** Subin Antony Jose, Antonia Evtimow, Pradeep L. Menezes

**Affiliations:** Department of Mechanical Engineering, University of Nevada, Reno, NV 89557, USA; subinj@unr.edu (S.A.J.); aevtimow@unr.edu (A.E.)

**Keywords:** 3D bioprinting, bioinks, tissue engineering, organ-on-a-chip, hydrogel scaffolds

## Abstract

Three-dimensional (3D) bioprinting has rapidly emerged as a transformative technology at the interface of biomedical engineering and regenerative medicine. By enabling the spatially controlled deposition of living cells, biomaterials, and bioactive molecules, it offers an unprecedented potential to fabricate functional tissues and potentially whole organs in the future. This review explores recent advances in bioprinting materials, processes, and applications, emphasizing the integration of bioinks, printing methods, and mechanical design principles that underpin tissue functionality. Natural and synthetic biomaterials such as hydrogels (e.g., collagen, alginate), polyethylene glycol (PEG), and polyesters like PLGA are evaluated in terms of biocompatibility, printability, and degradation behavior. Key bioprinting modalities, including extrusion, inkjet, and laser-assisted bioprinting, are compared based on printing resolution, cell viability, and scalability. Structural considerations such as scaffold architecture, mechanical stability, and biomimetic design are discussed in relation to native tissue mechanics and requirements. The review also surveys emerging applications in tissue engineering (e.g., bone, cartilage, skin replacements), organ-on-a-chip systems for drug testing, and patient-specific implants, while addressing persistent challenges such as standardization of biofabrication, regulatory and ethical considerations, and manufacturing scale-up. Finally, future trends, including the integration of artificial intelligence (AI) and robotic automation, multi-material and four-dimensional (4D) bioprinting, and the maturation of personalized bioprinting strategies, are highlighted as pathways toward more autonomous and clinically relevant bioprinting systems. Collectively, these developments signify a paradigm shift in how biological constructs are designed and manufactured, bridging the gap between laboratory research and clinical translation.

## 1. Introduction

Once confined to the realm of science fiction, 3D bioprinting has quickly evolved into a transformative technology at the intersection of engineering, biology, and medicine. The ultimate vision of bioprinting is to create complex organs on demand, such as printing a human heart for transplantation. Still, current technology has not yet reached that level of sophistication. Additive manufacturing (AM) itself dates back to 1984, when Charles Hull invented stereolithography (SLA) as a method for building objects layer by layer from photopolymer resin [1,2]. Bioprinting emerged in the decades thereafter as a natural progression, adapting 3D printing techniques to deposit living cells and biocompatible materials [3]. A major motivation for 3D bioprinting in biomedical science has been the limitation of traditional 2D cell cultures. Since the early 20th century, flat cell culture has been commonplace due to its simplicity, but it fails to replicate the complex 3D microenvironment of living tissues [4,5]. This discrepancy means that cells in 2D often do not behave as they would in vivo, making drug testing and disease modeling in 2D less predictive of clinical outcomes. Three-dimensional bioprinting addresses this problem by enabling the construction of tissue-like architectures where cells can interact in all dimensions, more closely mimicking native tissue structure and function [6,7].

### 1.1. Historical Milestones

**Figure 1:**
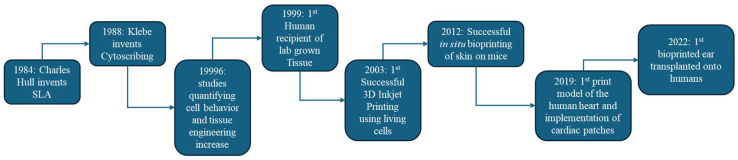
Timeline of Significant 3D Bioprinting Events.

The field of bioprinting has been punctuated by a series of important breakthroughs, as illustrated in Figure 1. In 1988, Robert Klebe demonstrated one of the first “bioprinting” concepts with a method called cytoscribing, using a modified inkjet printer to deposit patterns of fibronectin (an adhesive protein) onto collagen substrates, and seeding cells on these protein templates [8]. By stacking those cell-laden sheets, Klebe created simple 3D tissues, albeit needing manual assembly and reinforcement with collagen glue. In 1996, Ramsey Foty and colleagues provided critical insights for scaffold-free bioprinting by showing that embryonic cells behave like liquid droplets and will self-assemble due to surface tension and differential cell adhesion [9]. This finding (the differential adhesion hypothesis) suggested that if cells are placed in proximity, they can fuse and reorganize into tissues without a solid scaffold, an idea that later inspired the printing of cell spheroids as bioink. In 1999, a dramatic proof-of-concept for tissue engineering was achieved by Atala’s team, who engineered lab-grown bladders and successfully implanted them into patients. They cultured the patient’s own cells on a hand-sewn biodegradable scaffold and then reattached the engineered tissue in the bladder, illustrating clinical feasibility for engineered organs (though this procedure did not use a computerized printer, it set the stage for bioprinting by highlighting the importance of cells + scaffolds for organ regeneration) [10]. The first true 3D bioprinter was unveiled in 2003, when Chris Wilson and colleagues modified a standard Hewlett-Packard inkjet printer to dispense living cells as “ink” [11]. They printed both biomolecules (e.g., the word “Biotin” spelled in a bioactive protein) and viable cells in predefined patterns. Notably, the printed endothelial cells remained alive, and the patterns remained intact, proving that inkjet-based bioprinting could position cells without killing them. In 2012, bioprinting moved into the in vivo realm with Atala’s group using a portable inkjet bioprinter to deposit skin cells directly onto wounds in a mouse model, achieving accelerated wound closure with a layered skin construct printed right on the animal [12]. This demonstrated the potential of bioprinting for on-site tissue repair and wound healing. More recently, in 2019, researchers at Tel Aviv University bio-printed a miniature heart complete with tiny blood vessels, using a patient-derived cellular bioink. While only the size of a rabbit’s heart and not capable of full function, this achievement showed that multiple cell types (cardiac muscle cells, blood vessel cells) could be printed together in an organ proxy with internal chamber geometry [13]. And in 2022, the company 3DBio Therapeutics announced the first successful transplantation of a 3D-bioprinted human ear (composed of living cartilage cells) into a patient with microtia, yielding a living ear that cosmetically and biologically matches the patient’s other ear [14]. Each of these milestones, from printing basic cell patterns and simple tissues to clinically implanting bio-printed constructs, has expanded the scope of what bioprinting can achieve and foreshadows the eventual printing of fully functional organs.

### 1.2. Current Efforts

**Figure 2:**
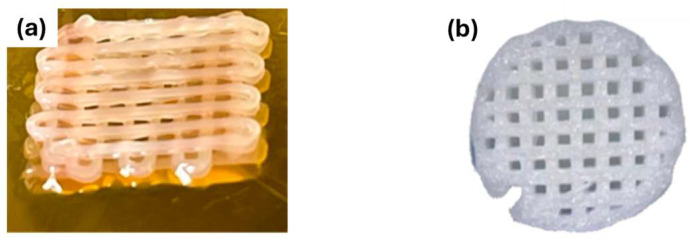
Representative bio-printed constructs for regenerative medicine applications: (**a**) co-printed alginate/gelatin hydrogel integrated with a supportive scaffold for cartilage regeneration, and (**b**) three-dimensional (3D) printed PCL/30HA composite scaffold. Reproduced from [16], Biomimetics, MDPI.

Although printing entire, fully functional organs (like a human-sized heart or kidney) remains a long-term goal, the current applications of 3D bioprinting are broad and impactful. Bio-printed tissues are increasingly used in regenerative medicine for manufacturing grafts and implants. For example, printed bone or cartilage scaffolds laden with stem cells can be used to repair orthopedic defects, and bio-printed skin patches are being developed for treating burns and chronic wounds [15]. Figure 2 illustrates representative examples of such applications, including bio-printed constructs designed for cartilage regeneration and composite scaffolds developed for subchondral bone repair.

In the realm of personalized implants, bioprinting allows the creation of patient-specific shapes, such as an auricular (ear) cartilage implant matching a patient’s anatomy, or a section of trachea printed to fit precisely in a particular patient, which can then mature into functional tissue after implantation [17]. In parallel, bioprinting is revolutionizing in vitro models. 3D-bioprinted cell constructs are used as more realistic models for drug testing, disease modeling, and toxin screening, sometimes referred to as “organ-on-a-chip” systems when combined with microfluidics [18,19]. For instance, a mini-liver tissue or tumor model can be printed with multiple cell types to test drug toxicity or to study cancer behavior in an environment that simulates the 3D interactions of real tissue better than a flat Petri dish culture [20]. Additionally, bioprinting has applications in creating surgical training models and prosthetics. Surgeons can practice with the bio-printed models of patient organs (like a bio-printed kidney phantom with tissue-like consistency), and researchers are exploring hybrid bioprinting to produce prosthetic devices that interface with the body (for example, a bio-printed interface with cells that encourages nerve integration on a bionic limb) [21,22]. Since most bio-printed constructs so far are smaller in size (millimeters to a few centimeters) and relatively simple, using them as research tools and specialized implants is a practical, near-term focus, laying the groundwork for tackling more complex organs in the future.

This paper provides a comprehensive examination of the field of 3D bioprinting. Section 2 outlines the fundamental principles of bioprinting, including the typical workflow (from pre-print cell preparation to post-print tissue maturation) and key printing methodologies. Section 3 surveys the landscape of bioink materials, distinguishing between natural polymers (like alginate, collagen) and synthetic polymers (like PEG, PLGA), and discusses their mechanical and biological properties along with strategies to combine materials for optimal performance. Section 4 details the major bioprinting techniques (extrusion, droplet-based, light-based, and laser-assisted), explaining their mechanisms, advantages, and limitations for maintaining resolution and cell viability. Section 5 covers structural and mechanical design considerations in bioprinting, such as scaffold geometry, pore size, mechanical strength requirements, and the need to mimic the extracellular matrix environment. Section 6 reviews current and emerging applications of 3D bioprinting across tissue engineering, organ-on-chip systems, and customized medical devices, with examples of recent successes. Section 7 discusses the challenges and limitations still facing the field, for example, achieving vascularization, ensuring reproducibility and regulatory compliance, and scaling up larger tissue volumes. Section 8 explores the future outlook, including how convergence with AI, robotics, and new materials could address these challenges and push bioprinting toward clinical reality. Finally, Section 9 concludes with an outlook summarizing how ongoing innovations are accelerating the transition of bioprinting from experimental setups to mainstream medical practice.

## 2. Fundamentals of 3D Bioprinting

In simple terms, 3D bioprinting uses biomaterials (often laden with living cells) to fabricate structures layer by layer that mimic tissues and organs. The general bioprinting process can be divided into three main stages: (1) Pre-print preparation, (2) the printing process, and (3) post-print maturation. Each stage has unique requirements to ensure that the final construct is viable and functional.

### 2.1. Design and Pre-Processing

The bioprinting workflow begins with digital design of the target tissue structure. This often involves using medical imaging (such as CT or MRI scans) of a patient’s anatomy to create a 3D model of the desired shape [23]. The model is then converted into a standard format (STL file) and “sliced” into discrete layers using computer-aided manufacturing software, generating a G-code toolpath that instructs the printer where to deposit material layer by layer [24]. At the same time, the bioink preparation takes place. This involves formulating the biomaterial that will be printed and incorporating cells into it. Cells are typically obtained from a donor tissue or stem cell source and expanded in culture to sufficient numbers (often in an incubator at 37 °C with controlled CO_2_ and nutrients). The biomaterial (such as a hydrogel solution) is mixed gently with the cells to create a printable cell suspension. Depending on the printing approach, the bioink’s properties (viscosity, gelation mechanism, etc.) are tuned appropriately [25]. For example, a bioink for extrusion printing might be a gelatin–alginate hydrogel that remains fluid in the printer cartridge but can rapidly solidify upon dispensing (through cooling or ionic crosslinking), whereas a bioink for laser-assisted printing might need to be a thin layer applied to a donor slide. Throughout this preparation, maintaining sterility is crucial. Prints are often performed in sterile enclosures or biosafety cabinets, and the bioink components are filtered or UV-sterilized in advance to prevent contamination of the living construct.

### 2.2. Bioprinting Process (Layer-by-Layer Fabrication)

With the design and bioinks ready, the actual printing is carried out by one of several methods. In all cases, the printer deposits successive layers of cells and material according to the predefined 3D pattern. Common bioprinting modalities include extrusion-based printing, droplet-based (inkjet) printing, light-based printing, and laser-assisted printing. Extrusion bioprinters extrude continuous filaments of bioink through a nozzle to draw each layer (similar to fused deposition modeling in plastic 3D printing). Inkjet or other drop-on-demand printers eject tiny droplets of bioink only where needed, achieving patterns by printing many small drops that coalesce. Stereolithography-inspired bioprinters use UV or visible light to photopolymerize cell-laden resin in specific regions, forming solid hydrogel scaffolds layer by layer within a liquid bath. Laser-assisted bioprinting uses pulsed laser energy to eject microdroplets of bioink from a donor surface to a collector, allowing nozzle-free, high-resolution deposition [26,27,28]. Bioprinters often include on-the-fly crosslinking or curing mechanisms, meaning that the bioink is stabilized immediately during or directly after deposition to preserve the printed geometry. Because most bioinks are initially in a liquid or semi-liquid state to allow extrusion or droplet formation, they would otherwise spread or collapse before solidifying. For instance, an extrusion printer may integrate a UV or visible-light source to photopolymerize methacrylated hydrogels (e.g., GelMA) layer-by-layer as they are printed [29]. Similarly, inkjet-based systems can deposit a crosslinking agent, such as calcium chloride, onto alginate either concurrently or immediately after printing each layer, to induce ionic gelation [30]. These strategies help maintain shape fidelity, improve structural stability, and enable the fabrication of multi-layered constructs.

A critical consideration during printing is maintaining cell viability: printing is preferably done in a temperature-controlled environment (commonly 20–37 °C, depending on the bioink) and completed as quickly as possible to limit the time cells spend outside an incubator [31]. Proper rheological properties of the bioink (e.g., shear-thinning behavior) allow it to flow through needles or nozzles under pressure yet retain its shape once deposited. Attention is also given to the porous structure of the printed object; typically, lattice or grid patterns are printed rather than completely solid masses, so that channels and pores exist for diffusion of nutrients and oxygen to the cells in the interior of the construct [32].

### 2.3. Post-Processing and Tissue Maturation

After printing, the freshly printed construct (often called a “pre-tissue” or a “construct”) is not yet a mature tissue. It usually requires a period of incubation and conditioning to develop the desired biological functions. First, any necessary crosslinking or curing of the biomaterial is completed. For example, a hydrogel may be chemically crosslinked by adding a crosslinker solution, or a temperature-sensitive gel may be cooled to strengthen it [31]. The construct is then transferred into a sterile container with cell culture medium and kept in an incubator to allow cells to proliferate, migrate, and deposit extracellular matrix (ECM). This stage can range from hours to weeks, depending on the application. In some cases, bioreactors are used to provide physical stimuli during maturation—such as perfusing media through vascular channels in a printed liver tissue, or applying mechanical loading or electrical stimulation to a printed cardiac patch—to encourage the cells to develop appropriate functional properties (e.g., muscle cells aligning and starting to contract synchronously, or cartilage cells producing collagen and proteoglycans for stiffness) [33]. Throughout post-processing, maintaining biocompatibility is essential. The materials should not elicit toxic or inflammatory responses, and any degradation of scaffolds should occur at a rate conducive to tissue formation (for instance, a scaffold might slowly biodegrade over weeks as new tissue matrix is produced). Care is also taken to avoid contamination; constructs are typically incubated in sterile plates or chambers. By the end of this stage, the goal is to have a tissue construct with sufficient cellular organization and extracellular matrix that it can perform the intended function or be implanted if needed.

**Figure 3:**
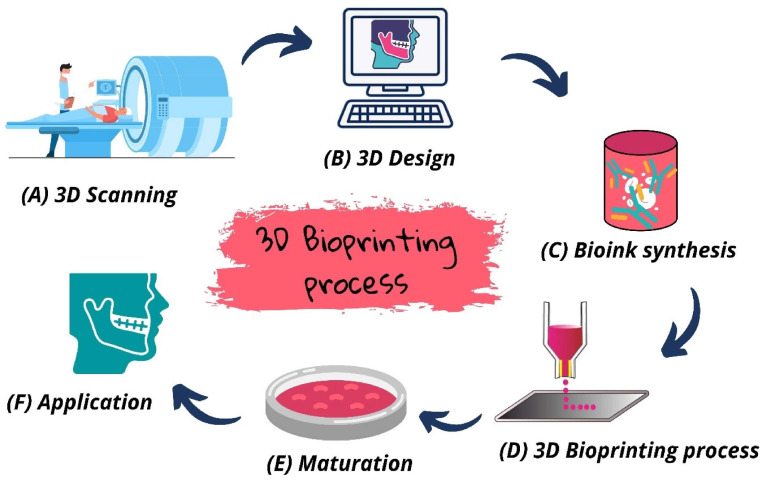
Generalized Workflow of the 3D Bioprinting Process—from design to post-culture. Key steps: imaging/CAD design, bioink formulation with cells, layer-by-layer deposition via chosen method, crosslinking stabilization, and bioreactor incubation. Reproduced from [35], Journal of Functional Biomaterials, MDPI.

It is worth noting that the entire bioprinting process must be carried out in a way that preserves cell health and replicates key aspects of the tissue’s native environment. For example, bioinks and crosslinking methods are chosen to be as gentle as possible on cells (e.g., using temperature changes or ionic crosslinking instead of harsh chemicals when feasible). Additionally, process monitoring is sometimes employed: advanced bioprinters may include imaging systems to check each layer (ensuring accuracy of deposition) or sensors to measure parameters like dispensing pressure, which can help in optimizing conditions to maximize cell viability and print fidelity [34]. Figure 3 outlines the general bioprinting workflow from design through maturation. Ultimately, the fundamentals of bioprinting combine principles of manufacturing (precise spatial control) with principles of tissue engineering (providing biological cells and signals), making it a uniquely interdisciplinary endeavor.

## 3. Materials for 3D Bioprinting

A bioink is a biocompatible formulation of cells with a matrix material (usually a polymer solution or gel) that can be printed to form a tissue construct. Choosing the right bioink is critical because it must simultaneously meet often competing requirements. It should be easily printable (appropriate viscosity, yielding smooth flow or droplets and then solidifying reliably), but also biologically conducive (supporting cell viability, adhesion, and differentiation) and, for implants, biodegradable with a controllable rate. Bioink materials are broadly classified into natural polymers, synthetic polymers, and composites (blends of multiple components, sometimes with added nanoparticles or fibers for reinforcement) [36].

The most common base materials for bioinks are hydrogels, a water-swollen polymer network that has tissue-like consistency. Hydrogels are attractive because of their extremely high-water content (often 90–99% water), which is like the native extracellular matrix (ECM) and allows diffusion of nutrients and oxygen to encapsulated cells. They also tend to be soft and flexible, which is compatible with cell movement and growth [37]. The ECM itself (the non-cellular scaffold of tissues) is largely hydrogel-like, composed of fibrous proteins (like collagen) and sugars (glycosaminoglycans) that retain water and present biological cues. Ideally, a bioink tries to mimic some aspects of the ECM, such as maintaining hydration, providing cell attachment sites, and having appropriate stiffness [38].

### 3.1. Natural Polymers

These are biomolecules derived from living organisms, such as polysaccharides and proteins, and they inherently tend to be biocompatible and often bioactive (cells have evolved to interact with them). Common natural polymers used in bioinks include:

(a) Agarose

Agarose is polysaccharide extracted from seaweed that has a unique thermo-reversible gelation behavior. It dissolves in hot water and forms a gel upon cooling below ~30 °C. Agarose gelation does not require any chemical crosslinker; its chains self-assemble into a network as the temperature drops. This rapid thermal gelation can be advantageous for printing, as agarose can be printed warm (fluid) and will solidify as it cools, giving good initial shape fidelity. It is also fairly strong for a hydrogel, helping printed structures hold their form [39,40]. However, agarose lacks cell-adhesive motifs (cells do not naturally bind to pure agarose) and its gelation temperature is below body temperature, meaning that keeping it solid and cell-laden at 37 °C can be challenging without additional support. Due to minimal biological cues, agarose bioinks are often used as a sacrificial material or as a supporting matrix rather than the primary cell carrier; for instance, agarose might be printed as a temporary support that is later melted away, or combined with a more cell-friendly polymer [41]. Studies show that while agarose itself is biocompatible, cells encapsulated in pure agarose may survive but remain round and do not spread or attach well. To address this, agarose is frequently blended with other polymers or functionalized with adhesion peptides if it is to serve as a long-term scaffold [42].

(b) Alginate

Alginate is a polysaccharide derived from brown algae, composed of mannuronic and guluronic acid units. Alginate is popular in bioprinting because it gels ionically in the presence of divalent cations like calcium (Ca^2+^). A liquid sodium alginate solution can be rapidly crosslinked into a gel by contacting it with Ca^2+^ (for instance, by printing the alginate into a calcium chloride bath or spraying Ca^2+^ solution onto deposited layers). This gelation is quick and cell-friendly (no harsh chemicals involved), and the gel stiffness can be tuned by alginate concentration and Ca^2+^ dose. Alginate hydrogels also tend to be stable and retain shape, making them excellent for maintaining printed structures [43]. On the downside, like many plant polysaccharides, alginate lacks sites for cell adhesion (cells encapsulated in pure alginate often remain spherical and can gradually lose function). As a result, alginate bioinks are commonly used in combination with more bioactive components. For example, mixing alginate with gelatin (denatured collagen) provides cell-binding motifs from the gelatin while alginate provides mechanical support and fast gelation; such blends allow printing of structures that are immediately stable and cell-responsive. In terms of mechanics, alginate gels can be adjusted: higher concentration or high guluronic content yields stiffer gels, whereas more mannuronic content yields softer, more elastic gels [44]. After printing, an alginate scaffold can be maintained as a permanent matrix or, if needed, it can be partially degraded later by chelating the Ca^2+^ (to open space for tissue growth). The biocompatibility of alginate is generally good (many cells show high viability in alginate), but to promote cell spreading and tissue development, alginate-based bioinks often include cell-adhesive peptides (such as RGD sequences covalently bound to alginate) or are used as part of a composite bioink [45].

(c) Chitosan

Chitosan is a polysaccharide derived from chitin (found in crustacean shells and fungi). Chitosan is interesting due to its antibacterial properties and biodegradability. It carries a positive charge in acidic conditions, which can disrupt bacterial cell membranes. Chitosan-based scaffolds can help reduce infection risk. Chitosan can form hydrogels, typically via pH-dependent solubility or by ionic crosslinking with multivalent anions [46]. It is soluble in mildly acidic water (pH ~6 or below) and solidifies when the pH is raised or when mixed with polyanions or crosslinkers. One challenge is that the physiological pH (~7.4) causes chitosan to precipitate, so printing with chitosan often involves keeping it in an acidic solution and then neutralizing it after printing—conditions that must be controlled to prevent harming cells. Chitosan alone gels somewhat slowly and yields relatively weak hydrogels, so it is frequently combined with faster-gelling agents (for instance, chitosan/alginate mixtures can gel by ionic crosslinking, as alginate provides the crosslinkable sites for Ca^2+^) [47]. Like alginate, pure chitosan lacks specific cell adhesion sequences and thus typically requires the addition of gelatin, collagen, or ECM components to support cell attachment and proliferation. Its mechanical strength is low on its own, but its presence in a composite can improve viscosity and printability. Chitosan is often investigated for cartilage tissue engineering and wound healing applications, where its antibacterial nature and support for cell differentiation (chondrocytes have been shown to redifferentiate in chitosan hydrogels) are beneficial [48].

(d) Cellulose (and its derivatives)

Cellulose itself is a fibrous, water-insoluble polymer (the primary component of plant cell walls). However, cellulose derivatives like methylcellulose, hydroxyethyl cellulose, and nanofibrillated cellulose are used in bioinks because they are water-soluble or can form hydrogels and significantly improve the rheological properties of inks. For example, adding a small amount of nano-cellulose fibers to a bioink can greatly increase its viscosity at rest and yield stress, which helps the bioink hold its shape after printing (acting as a kind of reinforcement). Methylcellulose can gel upon warming and is sometimes used to temporarily thicken bioinks during printing [49]. Cellulose-based materials are biocompatible and chemically stable, but not enzymatically degradable in the human body (humans lack cellulase enzyme), so if they are used, they often remain as an inert component or are intended to be non-permanent but removed by perfusion or other means [50]. They also do not support cell adhesion on their own. Thus, like other inert polymers, cellulose additives are usually combined with more bioactive polymers. One successful approach has been mixing nanocellulose with alginate for printing cartilage. The nanocellulose provides mechanical strength and shear-thinning behavior for printability, whereas alginate (with perhaps a bit of gelatin added) provides the gelling mechanism and a matrix for cells. Such bioinks have shown good shape fidelity and have been used to print ear or meniscus shapes with adequate mechanical properties for handling [51].

(e) Collagen

Collagen is ahe most abundant protein in the human body and a primary component of ECM for many tissues. Collagen type I self-assembles into fibrils at neutral pH and 37 °C (starting from an acidic soluble solution) to form a gel. Collagen is inherently biocompatible and bioactive. Cells readily attach to collagen via integrin receptors, and this can promote cell differentiation and tissue organization. Collagen gels are often used to embed cells for bioprinting, but native collagen has a few drawbacks: it has a low viscosity in the monomeric form (difficult to print unless kept cold and concentrated), relatively slow gelation (minutes to hours as it warms up and neutralizes), and the final gel is very soft and fragile [39]. To use collagen in bioprinting, one strategy is to print it at a low temperature or in a dispersed form and then induce gelation. Another strategy is to chemically modify collagen to allow faster crosslinking. Because of collagen’s excellent cell-friendly nature, it is extensively used in combination with other materials: for instance, collagen may be mixed with alginate (the alginate provides immediate mechanical support via ionic gelation, then collagen later polymerizes to add bioactivity), or with fibrin (another protein that polymerizes via enzyme action). Crosslinkers like genipin or glutaraldehyde can also be used to strengthen collagen gels, but those chemicals can be cytotoxic, so they must be used carefully and often washed out thoroughly. Overall, collagen is indispensable for ensuring a bioink’s biological affinity to cells, but often needs help from other polymers or crosslinking methods to be print-friendly [48].

(f) Gelatin

Gelatin is a denatured form of collagen, obtained by thermal or chemical breakdown of collagen (for example, from animal connective tissue). Gelatin shares many of collagen’s benefits. It has numerous cell-adhesive RGD sequences and natural degradation sites. But unlike collagen, it does not self-assemble into fibrils. Instead, gelatin behaves as a thermoresponsive polymer. It is liquid at warm temperatures (above ~30 °C) and forms a gel upon cooling (below ~25–30 °C) due to the formation of partial helical structures [48]. Regular gelatin gels melt at body temperature, so a common approach is to use GelMA (gelatin methacryloyl), which is gelatin chemically modified with photopolymerizable groups (methacrylates). GelMA can be crosslinked with UV light in the presence of a photoinitiator, forming a stable network even at 37 °C. This has made GelMA one of the most popular bioink materials, as it offers biological cues from gelatin and tunable mechanical properties via controlled crosslinking intensity/time [52]. Even unmodified gelatin is useful in bioprinting. It can be printed at a moderately low temperature to maintain a gel state during deposition (for example, printing in a cold environment or onto a cooled plate), a method used in some support bath bioprinting techniques. However, without chemical crosslinking, gelatin will eventually dissolve at 37 °C, so it is mainly used either in combination with other permanent networks or crosslinked into GelMA. GelMA, by varying the degree of methacrylation and concentration, can achieve a range of stiffnesses to match soft tissue properties and has been used to print cartilage, cardiac tissue, and vascular structures with good cell outcomes [53,54].

(g) Decellularized Extracellular Matrix (dECM)

This refers to material derived from native tissues by removing all the cells, leaving behind the natural ECM composed of proteins (collagens, laminin, fibronectin, etc.) and glycosaminoglycans, along with bound growth factors. dECM from tissues (like heart, liver, cartilage, etc.) can be processed into a powder and then into a hydrogel that retains many of the biochemical cues of the original tissue. dECM bioinks are attractive because they inherently provide a biomimetic environment. Cells printed in a dECM from cardiac tissue, for example, receive some of the same signals they would in a native heart matrix. Studies have shown that cells often display enhanced function and maturation in dECM-based bioinks compared to simple single-polymer gels. The challenge is that dECM gels can be very soft and slow to gel. They often require a combination of temperature and pH change to gel (many are collagen-based) and may need additional crosslinking to be handleable. Therefore, dECM bioinks are frequently mixed with another polymer (for instance, a small percentage of genipin to crosslink the proteins, or blended with GelMA to allow UV curing) [41]. Despite these challenges, dECM bioinks have been successfully printed for applications like myocardial patches and liver tissue models, where their rich composition promotes appropriate cell behavior. Decellularization also reduces the immunogenicity of the material (since cells and DNA are removed), though some risk remains if any residual immunogenic molecules are present. As a point of note, since dECM is sourced from tissues, its composition can vary, and reproducibility is an issue unless the sourcing and processing are well-controlled [39].

### 3.2. Synthetic Polymers

These are man-made polymers (commonly used in medical materials) utilized in bioinks primarily to impart mechanical strength, structural support, and consistent properties. Synthetic polymers generally lack specific biological signals (cells often do not naturally adhere to them, hence they are considered “bioinert” or at best, not actively instructive), but they offer tunability and robustness. Key synthetic polymers in bioprinting include:

(a) Thermoplastics (e.g., PCL, PLA, PLGA)

These are polyester polymers like poly (ε-caprolactone) (PCL), poly (lactic acid) (PLA), poly (glycolic acid) (PGA), and their copolymer PLGA. They are widely used as scaffold materials in regenerative medicine due to their biodegradability via hydrolysis and established use in FDA-approved implants. In bioprinting, these polymers are often printed in molten form (at 80–150 °C) as supporting frameworks (this is sometimes called melting deposition manufacturing or a hybrid bioprinting approach). For example, a stiff PCL grid can be printed to provide a load-bearing structure, and then cell-laden hydrogel bioink is printed into the spaces of the grid. PCL and PLA provide excellent shape fidelity and mechanical strength, exhibiting elastic moduli of approximately 0.2–0.4 GPa (PCL) and 1–3 GPa (PLA), compared with soft hydrogels whose moduli typically fall in the kPa to low-MPa range [55,56,57]. However, printing them involves high temperatures that would kill cells, so these thermoplastics are printed in an acellular fashion, typically in a different printhead from the cell ink (or printed first, then seeded with cells or filled with cell gel). They also degrade slowly (PCL can take years to fully degrade, while PLA/PLGA in the order of months, depending on molecular weight and copolymer ratio), and their byproducts can be acidic (PLA and PLGA release lactic and glycolic acid). Thus, while they contribute mechanical integrity, they must be used in moderation or with design considerations to avoid harm to cells. In summary, thermoplastics are more commonly used as a printed scaffold framework in conjunction with cell-laden hydrogels, rather than being directly mixed with cells in a single bioink. This strategy of hybrid printing capitalizes on the strengths of both types of materials [41].

(b) Polyethylene Glycol (PEG)

PEG is a hydrophilic synthetic polymer that is often used in hydrogel form. It is considered bioinert (cells do not adhere to pure PEG), but it is highly tunable; one can introduce functional groups for crosslinking and attach peptides for bioactivity. PEG-based hydrogels are typically formed by chemical or photochemical crosslinking of multi-armed PEG molecules (e.g., PEG diacrylate). The advantages of PEG are its predictable and controllable properties. By adjusting molecular weight and crosslink density, one can obtain a desired stiffness and degradation rate. PEG hydrogels also have excellent transport properties (being hydrophilic) and can be made degradable by incorporating ester linkages (e.g., PEG-co-lactic acid copolymers). For bioprinting, PEG is frequently used in the form of PEGDA (PEG diacrylate) or PEG-fibrinogen, etc., which can be photo-crosslinked during or after printing. This permits high-resolution structural fixes. Because PEG lacks cell adhesion, researchers often tether cell-binding peptides (like RGD) onto PEG chains or encapsulate cells in a mixed PEG + bioactive matrix hybrid. For example, a bioink might use a PEG network for structural support, but also include fibrin or gelatin so that cells have something to grab onto. PEG can be non-degradable unless modified, so depending on the application, it is often engineered either to remain as a permanent but inert scaffold (like in some in vitro chips) or to degrade hydrolytically if used in vivo (by using PEG-co-polyester crosslinkers that break down). Overall, PEG’s role in bioinks is usually to provide a mechanically stable and tunable blank slate scaffold, which then needs to be biofunctionalized for cell culture [39].

(c) Pluronic F127

A triblock copolymer (PEO-PPO-PEO) that is commercially available and known for its thermosensitive behavior. Pluronic is a liquid at low temperature (4 °C) but forms a gel at body temperature (around 37 °C) at sufficient concentration. This reverse thermal gelation, along with Pluronic’s excellent shear-thinning properties, makes it very useful as a sacrificial bioink. For instance, Pluronic can be printed at room temperature as a support or a filler, and then later removed by cooling and dissolving it away (since it liquefies when chilled). It is often used to create channels. One can print Pluronic as a filament network within a structure, then flush with cold solution to leave hollow vascular-like channels. Pluronic is biocompatible in the short term, but is not usually left in constructs long-term. It is also non-degradable. Its main advantages are in the printing phase: it increases print fidelity and can act as a removable template. Pluronic can also be mixed with other inks to temporarily enhance viscosity during printing, then leach out. It is sometimes included in small amounts to make an ink more shear-thinning (since Pluronic’s viscosity drops dramatically under shear) [39,48].

Other synthetic polymers occasionally employed in bioinks include poly (vinyl alcohol) (PVA), which is water-soluble and can be chemically crosslinked (but requires cell-adhesive modification for cell culture), and self-assembling peptides or polypeptides that are designed to form gels. Each synthetic polymer on its own has specific mechanical profiles. For example, PCL is very flexible and slowly degrades, while PLA is more brittle and faster. By varying blends and molecular weights, one can achieve the desired scaffold longevity and stiffness. A summary of commonly used bioinks, including their polymer classification, solubility characteristics, crosslinking mechanisms, and key advantages and limitations, is presented in Table 1.

### 3.3. Composite Bioinks

In practice, many bioinks are composites, meaning they combine multiple materials to leverage the strengths of each while mitigating weaknesses. A classic example is mixing a natural polymer with a synthetic one, like alginate–gelatin, collagen–PEG, fibrin–Pluronic, etc. Natural polymers contribute bioactivity (cell adhesion sites, biological recognizability) while synthetic polymers contribute printability and mechanical support. Composite bioinks may also include particulate additives (fillers) such as bioactive nanoparticles, short fibers, or nanosheets to enhance certain properties [78]. For example, adding nano-hydroxyapatite particles (a ceramic found in bone) to a bioink intended for bone tissue can improve its stiffness and promote bone cell differentiation by releasing calcium/phosphate ions. Adding graphene oxide or carbon nanotubes can increase the mechanical strength of a soft hydrogel and introduce electrical conductivity, which is useful for cardiac or neural tissue engineering (where electrical signals are important). Incorporating vascular endothelial growth factor (VEGF) or other growth factors in microspheres inside a bioink is another composite strategy, providing slow release of signals to encourage vascularization after printing [79,80]. The different types of fillers used in bioink and composite biomaterial formulations, along with their primary functions and representative applications, are summarized in Table 2.

Composite bioinks often must be optimized empirically. The rheology (flow behavior) of a multi-component ink can be complex; sometimes, one polymer dominates the viscosity, while other times they synergistically increase yield stress. A desirable rheological profile for extrusion is shear-thinning with a high yield stress, meaning the ink is solid-like at rest (maintaining shape), but flows easily when shear (force) is applied in the nozzle. Many composite inks achieve this by combining a long-chain polymer (providing viscosity) with a smaller associative polymer or particles. For example, alginate (long flexible chains) plus nanocellulose (rigid rods) yields such behavior: at rest, nanocellulose forms a network that holds alginate in place; under shear, that network breaks down, and the alginate chains align and flow [78].

From a biological standpoint, composite bioinks aim to create a microenvironment that instructs cells while printing well. Hydrogels like PEG or alginate can be thought of as blank scaffolds that need “information” added. This information can come from blending in some dECM, adding a short peptide, or even encapsulating a second cell type (a composite in terms of cellular composition) to provide signaling. There is active research in creating “smart” bioinks that respond to the cell’s activity [92,93].

In summary, no single material currently meets all the needs of bioprinting. Therefore, combining materials is the norm: natural polymers to keep cells happy, synthetic polymers to aid fabrication, and strategic additives to enhance mechanics or bioactivity. The resulting composite bioinks strive to have: (a) suitable mechanical integrity during and after printing, (b) high cell viability and appropriate cell–matrix interactions, and (c) a controllable degradation profile if implantable. Progress in bioink development continues to be a linchpin of bioprinting advances, as creating the “ideal” bioink remains a challenge that requires balancing multiple trade-offs [94].

## 4. Bioprinting Techniques

**Figure 4:**
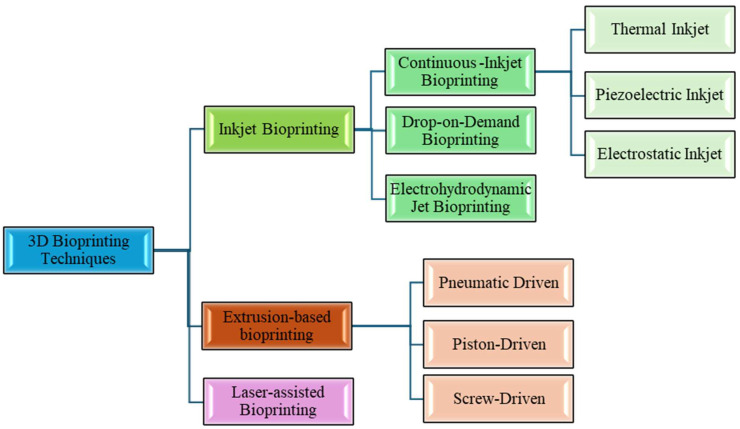
Classification of 3D Bioprinting Techniques.

Several distinct 3D printing modalities have been adapted for bioprinting, each with unique mechanisms to deposit cells and biomaterials in a spatially controlled manner. The primary bioprinting techniques are generally categorized as inkjet (droplet-based) bioprinting, extrusion-based bioprinting, laser-assisted bioprinting, and emerging hybrid or specialized methods. Each technique offers different strengths in terms of resolution, speed, cell handling, and allowable bioink properties. Figure 4 illustrates schematics of these major bioprinting approaches. In this section, we describe each technique, compare their capabilities (such as achievable feature size and cell viability), and note examples of use cases [95,96].

### 4.1. Inkjet and Droplet-Based Bioprinting

Inkjet bioprinting was one of the first methods developed, stemming directly from office inkjet printers but replacing ink with bioink. It is a form of drop-on-demand (DOD) bioprinting, where discrete picoliter droplets of bioink are ejected and deposited at specific locations. Inkjet bioprinters typically use either thermal or piezoelectric actuators to generate droplets [95]. The printing process is very fast (thousands of droplets per second can be generated), and resolution can be quite high; droplet diameters are in the order of 50–100 μm, and printed features can be of similar scale. However, bioinks for inkjet must be low-viscosity (≈3–20 mPa·s) so that they can form droplets and not clog the nozzles [97]. This typically limits inkjet to relatively diluted polymer solutions and smaller cell densities [96].

There are two main operational modes for droplet printing. They are continuous and drop-on-demand. In continuous inkjet, a continuous stream of liquid is broken into droplets by vibrations (Rayleigh–Plateau instability) and either directed to the substrate or into a waste reservoir by charging some droplets and using deflection plates. Continuous mode is less common in bioprinting because it is harder to sterilize and encapsulate, though it offers very high speed. Drop-on-demand inkjet, by contrast, creates droplets only when needed at specific locations, making it more controlled and gentler. Thermal inkjet DOD printers heat a tiny pocket of ink rapidly to create a bubble that pushes a droplet out of the nozzle, whereas piezoelectric DOD printers use a piezo crystal that flexes when voltage is applied, generating a pressure pulse that forces a droplet out [98]. Both can produce consistent ~50 μm droplets on demand. Biologically, studies have found that the brief thermal pulses (which can reach ~300 °C for a few microseconds in a very local spot) do not significantly affect cell viability in the droplet, presumably because the droplet itself does not overheat much. Piezoelectric printing does not introduce heat but does impose pressure waves; however, most mammalian cells tolerate these well if the bioink is formulated to avoid an amplitude too high. With careful parameter tuning, cell viabilities of 85–95% are routinely achieved with inkjet bioprinting immediately after printing [96,99].

A major limitation of inkjet bioprinting is the bioink viscosity requirement. Many desirable bioinks (like concentrated collagen or cell aggregates) are too viscous to be jetted through the tiny nozzles. Clog-free operation typically requires filtration of bioink to remove cell clumps or large ECM fibers. This restricts printable cell density. Inkjet-printed bioinks often have cell concentrations around 10^6^–10^7^ cells/mL. Also, because droplets are printed one by one, building large 3D structures can be time-consuming; inkjet is very fast per droplet, but it might need many layers of droplets to build thickness [100].

One extension of droplet printing in bioprinting is electrohydrodynamic (EHD) jet printing, which uses an electric field to induce extremely fine droplets or even continuous jets from a needle. EHD printing can achieve sub-10 μm resolution, essentially drawing ultrafine lines or droplets by leveraging electrostatic force to form a Taylor cone of the bioink at the nozzle tip. Researchers have used EHD to print micro-scale patterns of cells or biomolecules, but it is a more complex setup and not yet as widely used as piezo/thermal methods. Early results show EHD can maintain high cell viability (~90%) and place cells with very high precision, but it still shares the need for low-viscosity inks [101].

#### 4.1.1. Advantages

Inkjet bioprinting is valued for its high resolution and gentle handling of cells. The absence of continuous mechanical shearing (compared to extrusion) can be kinder to delicate cells. It is also fast in X–Y patterning; hence, one can cover a large area quickly with droplets. It is ideal for creating gradients of cells or biomolecules by using multiple printheads each loaded with different ink, and for printing things like microarrays for high-throughput experiments. Inkjet bioprinting has successfully been used for applications like printing skin, printing microvasculature within gels, and biomaterial patterning [100]. Additionally, inkjet bioprinting offers precise control over droplet volume and placement, enabling reproducible patterning at the microscale [102]. However, its applicability is generally limited to low-viscosity bioinks and moderate cell densities, which can constrain the range of printable materials.

#### 4.1.2. Limitations

The primary limitations are the narrow range of bioink viscosities that can be used and the difficulty in building up thick 3D tissues (since low-viscosity inks tend to be more liquid and droplets can merge or sag). If one tries to inkjet print many layers, the bottom layers may still be fluid, and cells can settle or diffuse, leading to less spatial accuracy in Z. To mitigate this, sometimes inkjet printing is done into a supportive medium. For example, printing droplets into a gelatin slurry that holds them in place (an approach combining inkjet with embedded printing concepts). Also, the print bed often must be kept cool, or the drops allowed crosslink as they accumulate, to avoid the construct just turning into a puddle. Another issue is that inkjet nozzles can clog if cell aggregates or undissolved material are present, thus requiring careful preparation of the bioink (often including filtering, which can reduce cell counts). Finally, living cells passing through a tiny nozzle have size constraints. Very large cells or cell clusters might not print well. Despite these challenges, when used appropriately, inkjet bioprinting achieves excellent feature fidelity for relatively planar or thin constructs and is a powerful tool for multi-material patterning on small scales [96,98].

### 4.2. Extrusion-Based Bioprinting

Extrusion bioprinting is currently the most widely adopted bioprinting method due to its versatility and ability to print a broad range of bioinks, including those with high viscosity and high cell density. In an extrusion bioprinter, the bioink (often a hydrogel or paste) is loaded in a syringe- or cartridge-like printhead and continuously pushed out through a nozzle to form a filament (strand) of material. The deposition system moves in X–Y–Z to lay down these filaments in the desired pattern (commonly a lattice or concentric pattern) that builds up layer by layer. After printing, the extruded filaments maintain their shape (assuming the bioink is formulated to be at least partly self-supporting or rapidly crosslinked), resulting in a 3D scaffold.

Extrusion systems can be driven in three primary ways: (a) pneumatic pressure, where compressed air forces the bioink out; (b) piston or plunger, where a mechanical plunger (motor-driven) pushes the bioink; or (c) screw-driven, where a rotating screw augers the bioink forward (like a miniature auger extruder). Each mechanism affects the bioink differently: pneumatic systems are easy to sterilize but can have a delay in start/stop and can compress air, leading to some “springiness” in flow; piston systems give direct control over volume but may have slower response; screw systems provide continuous feeding and work well with very viscous inks but introduce shear due to the screw motion. Many modern bioprinters use pneumatic or piston extrusion [96].

A huge advantage of extrusion is that it can handle bioinks from low viscosity (~30 mPa·s, like inkjet) up to extremely high viscosity (~10^7^ mPa·s, like a soft clay). This means even cell aggregates, tissue spheroids, or heavily loaded hydrogels (with 20–50% polymer content or high particle content) can potentially be extruded. It also allows printing of bioinks with very high cell concentrations, even tissue spheroids, as the “ink”. The trade-off is that resolution is lower: typically, extrusion nozzle inner diameters range from ~150–400 μm, and the printed filament width is in that ballpark or slightly larger (because the material might swell a bit after extrusion). Thus, feature sizes ~200–1000 μm are common. This is coarser than inkjet or laser-assisted printing, but still suitable for many tissue applications where multi-cell-thick features are acceptable (for example, small blood vessels are in the order of 100–500 μm).

During extrusion, cells experience significant shear stress as they travel through the needle, especially near the walls. Viability directly after extrusion printing often ranges from 40% to 90%, depending on factors like needle diameter, extrusion pressure/speed, and bioink composition. Typically, using larger nozzles, slower extrusion speeds, and more lubricious bioinks (e.g., with shear-thinning behavior that reduces stress at high shear rates) improves viability. For instance, encapsulating cells in a very soft hydrogel that flows easily will protect them better than printing cells in a highly viscous paste. Nonetheless, many cell types have been successfully printed via extrusion with high viability. For example, human mesenchymal stem cells in a collagen/alginate bioink retained ~85% viability and proliferative ability. It is often observed that cells might be slightly damaged but can recover within a day or two of culture post-printing, especially if provided a nurturing environment [98,103].

Extrusion printing often employs rapid crosslinking or gelation of the filament as it exits the nozzle to help it maintain shape. For example, a very common method is using coaxial extrusion for alginate bioinks: a needle-in-needle setup where the inner needle has alginate-cell suspension, and the outer needle flows a calcium chloride solution. As the two streams meet at the nozzle tip, the alginate instantly gels, yielding a cell-laden alginate thread that holds its form. Another approach is printing into a supportive material (such as a gelatin slurry or a yield-stress support bath) that holds the extruded filament in place until it solidifies fully—this is known as embedded bioprinting. A notable example is printing a complex vascular network by extruding a fibrinogen bioink containing cells into a gelatin-based support bath that provides stability until the fibrinogen is enzymatically crosslinked into fibrin [104].

Because extrusion can print high-viscosity materials, it is possible to include a lot of solid content like microfibers or particles, which can further impart immediate rigidity to the extruded filament. For instance, a bioink loaded with short cellulose fibers might come out almost as a paste that keeps its cylindrical shape perfectly without needing an external crosslink trigger, thus allowing freeform structures [105].

Extrusion bioprinting typically creates structures with internal strut or fiber spacings of a few hundred microns, which is convenient for tissue engineering because such pore sizes allow cell migration and nutrient transport. For example, a common printed scaffold pattern is a 0°/90° alternating grid (creating a mesh) with filaments ~400 μm thick and ~800 μm apart. This kind of structure has been used for bone and cartilage printing, among others. The filament can also be continuously varied (e.g., printing spiral or concentric rings for tubular structures like blood vessels). While single-nozzle extrusion prints one bioink at a time, many devices have multiple extruders, enabling multi-material extrusion. This allows, for example, printing a supportive synthetic polymer with one nozzle and a cell-laden hydrogel with another, or printing different cell types in different regions by swapping cartridges mid-print. The multi-nozzle capability, combined with the ability to deposit larger numbers of cells, means extrusion is often used to fabricate heterogeneous tissues. For example, interfacing soft and hard regions like osteochondral (bone–cartilage) constructs by printing a stiff biomaterial for the bone region and a softer hydrogel with chondrocytes for the cartilage region in one contiguous piece [106].

#### 4.2.1. Advantages

The key advantages of extrusion bioprinting are versatility and scalability. It can print very large constructs (many centimeters) because it is not limited by having to keep every part liquid; as long as the extruded material can support the next layer, you can keep building up. The hardware is relatively straightforward (syringe pumps or pneumatic controllers are widely available), and many researchers have converted standard 3D printers (used for plastics) into bioprinters by adding temperature control and sterile enclosures. Extrusion is compatible with a huge variety of bioinks, including those containing high cell densities, ECM components, or composite formulations, as discussed. Another advantage is that extrusion printing naturally creates a fibrous microstructure (the filaments), which in some cases is desirable as it can mimic fibrous tissues or guide cell alignment along the printed strands. For example, muscle or nerve cells tend to align along the printed lines of a fibrillar scaffold, aiding the formation of oriented tissue [107].

#### 4.2.2. Limitations

The main drawback is the lower resolution compared to inkjet or laser methods. Fine features below ~100 μm are difficult to achieve, which means microcapillary-sized channels are challenging to print directly (though some creative methods like coaxial extrusion can produce a tube with ~100 µm lumen). Also, cell stress is a concern, meaning optimizing the process to be gentle is an ongoing area of development (for example, using needle vibration or rotating to reduce stagnation points and ease flow has been tested to improve viability). Another limitation is the potential for inconsistent filament deposition if the bioink rheology is not ideal: under-extrusion can cause thin spots/gaps, and over-extrusion can lead to filament merging or blobs. The start/stop control can sometimes produce a droplet at the start or end of a line (this is mitigated by crisp control or sacrificial paths). Additionally, nozzle clogging can happen, especially when using very high cell densities or particulate-laden inks. Cells can aggregate and clog a tip, halting the print. Using larger nozzles or periodically agitating the cartridge can alleviate this [108].

Despite these issues, extrusion bioprinting remains the workhorse method for bioprinting, particularly for building volumetric tissues and implants. It is also relatively forgiving. Where an inkjet might fail to eject a droplet if conditions are off, an extrusion system might still push material through, albeit at a slightly different rate. Many of the landmark demonstrations of bio-printed tissues and organs (from printed cell-laden bone scaffolds to mini-organs) have used extrusion-based approaches because of this robustness.

### 4.3. Laser-Assisted Bioprinting (LAB)

Laser-assisted bioprinting uses a pulsed laser as a microengine to transfer bioink droplets to a substrate without a physical nozzle. The most common form is Laser-Induced Forward Transfer (LIFT) [109]. In LIFT bioprinting, one coats a thin donor slide with a layer of bioink (usually a thin film of cells in a liquid or light-sensitive gel) and backs it with a thin metal absorbing layer (like gold or titanium). A focused laser pulse (for instance, a nanosecond pulsed UV laser) is aimed at the metal layer from above (through the glass slide). The metal absorbs the laser energy and creates a high-pressure vapor bubble that ejects a tiny volume of the bioink directly below it, which travels downward and lands on the acceptor substrate positioned a short distance (e.g., 1 mm) below. By moving the laser focus in a pattern and firing pulses, one can effectively “print” droplets from the donor to specific spots on the collector. Each laser pulse can deposit a droplet in the order of 10–100 µm in diameter, with volumes in the picoliter to nanoliter range [109].

A key concern regarding LAB is the potential thermal damage to cells during laser exposure. In typical LIFT configurations, a pulsed laser is absorbed by a thin metallic layer (e.g., gold or titanium), leading to extremely high transient temperatures at the metal surface, often exceeding the metal’s boiling point and reaching several thousand degrees Kelvin. Importantly, experimental and numerical studies have demonstrated that this intense heating is highly localized and confined to a very thin micrometer-scale region immediately adjacent to the absorbing layer [110,111]. The thermal penetration depth into the bioink is minimal, and cells located beyond this narrow interfacial zone experience negligible temperature rise. Investigations using sensitive biological models, including yeast and mammalian cells, have shown that cell viability remains high because the dominant mechanism governing cell ejection is a rapid mechanical impulse rather than bulk thermal transfer. Consequently, LAB can achieve precise cell placement while maintaining post-print cell viability, typically exceeding 90%, provided that laser fluence is carefully optimized [112].

LAB is capable of very high-resolution printing, often cited at 20–50 µm for positioning accuracy, limited mostly by the laser focus spot size and the thickness of the donor bioink layer. It can also handle reasonably viscous bioinks on the donor (since there is no nozzle to clog); typically, donor inks might be a bit more fluid than extrusion inks to form a uniform film, but they can be loaded with cells at high density. One of the major advantages is nozzle-free printing, eliminating shear stress through a narrow orifice. This means even fragile cell types or bioinks with aggregates can be printed if the film is uniform. Moreover, multi-material printing is straightforward: one can set up different donor regions with different bioinks on the slide and thus print complex patterns of multiple cell types with high spatial control [113].

Laser-assisted printing has been shown to maintain very high cell viability, often 90% or above. The laser pulse is extremely brief and primarily heats the thin metal layer (most setups use a biocompatible sacrificial metal like gold or titanium that only needs to be ~50 nm thick). The cells themselves experience a thrust but not a significant temperature rise or other long exposure. The resulting droplet flight is through the air. Studies have printed delicate stem cells and even cell spheroids via LAB with good survival and differentiation post-printing. There is a limit to how high the laser fluence can go. If it is too intense, it can create a shockwave that might lyse cells, or it might ablate more vigorously and damage biomolecules. But within a well-tuned range, the process is gentle. Another benefit is that because there is no contact, sterility is easier (the donor can be a sterilized slide, and no nozzle means less contamination risk) [114].

#### 4.3.1. Advantages

Laser bioprinting offers excellent placement accuracy, the ability to print without a nozzle, and often high cell viability due to the non-contact nature. It is particularly good for complex multimaterial patterns at the microscale. For instance, making a microscale tissue where every 50 µm there is an alternate cell type, something extrusion could not achieve. It also does not rely on bioink being flowable in the traditional sense, just coat-able on a slide. That means, for example, one could print with extremely high cell concentrations or with bioinks that are more like a paste, if a thin layer can be prepared [115].

#### 4.3.2. Limitations

Downsides are that LAB is a more complex and expensive setup (requires laser, optics, precision stages, etc.) and typically prints in a serial fashion (one droplet per laser pulse), which, although quite fast (kHz pulse rates), can be slower than pushing out a continuous filament for large, simple structures. Building thick 3D structures by LAB can also be challenging: if you pile many droplets, the lower layer might still be fluid, and droplets could coalesce or splatter. Often, LAB is used to pattern cells on a surface or within a thin layer of gel rather than to build up large solid objects. Another limitation is access to the bioink layer. You must prepare a homogeneous film of bioink on the donor for uniform droplet ejection. If the bioink dries or does not spread evenly, ejection can become inconsistent. Thus, controlling the donor film quality is an extra step not needed in nozzle-based systems [115].

**Figure 5:**
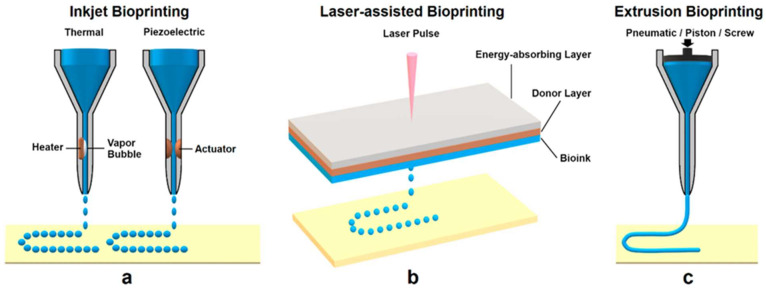
Common bioprinting techniques: (**a**) inkjet bioprinting, (**b**) laser-assisted bioprinting, and (**c**) extrusion bioprinting. Reproduced from [101], Polymers, MDPI.

Overall, LAB excels for high-resolution bioprinting tasks, such as printing micro-tissue architectures and complex co-culture patterns, whereas extrusion excels at bulk tissue fabrication, and inkjet sits somewhat in between (fast 2D coverage with moderate resolution). In practice, some research setups combine technologies. For instance, one might extrude a bulk hydrogel and then use laser bioprinting to “decorate” it with cells of different types in precise locations, achieving a hybrid of macro- and micro-scale organization. Figure 5 shows the schematic of the common bioprinting techniques.

In addition to thermal considerations, another limitation is the formation of nanoparticles originating from the laser-irradiated absorbing metallic layer. During repeated laser pulses, nanoscale metal fragments or vapor-condensed nanoparticles may be generated and entrained within the ejected bioink droplets. These nanoparticles have the potential to interact with transferred cells, influencing cellular behavior through mechanisms such as oxidative stress, altered adhesion, or unintended bioactivity [116]. While some studies suggest that nanoparticle concentrations are generally low and may not significantly compromise short-term cell viability, their long-term biological effects remain insufficiently understood. As a result, careful selection of absorbing layers, optimization of laser parameters, and the use of alternative sacrificial layers or dynamic release coatings have been proposed to mitigate nanoparticle formation. Addressing these issues is essential for advancing LAB toward reliable, reproducible, and clinically relevant bioprinting applications [117].

### 4.4. Emerging and Hybrid Bioprinting Techniques

Beyond the main methods above, various hybrid or novel bioprinting approaches are being developed. Micro-extrusion + UV systems can print photocurable bioinks with extremely high fidelity (sometimes called stereolithography bioprinting if using a projection). For example, digital light processing (DLP) bioprinters project entire images to cure a whole layer of a cell-laden photopolymer at once. This allows printing complex shapes with microscale features internal to the structure, but viability can be an issue if exposure times are long. 4D bioprinting, printing structures that change over time or with stimuli, is an exciting offshoot, where the printed tissue can later mature or be triggered to transform [118,119].

Another niche method is acoustic bioprinting, where acoustic forces are used to eject droplets from a liquid or to position cells. This is still in early research, but holds promise for nozzle-free printing like laser, but potentially cheaper hardware [120].

Embedded bioprinting has also become quite popular. It essentially allows use of extrusion or droplet printing to create complex shapes by printing into a support bath that holds everything in place until solidified. For example, freeform printing of branching vascular channels inside a matrix has been achieved by extruding a fugitive ink (like Pluronic or gelatin) into a gel support; the printed network does not collapse because the surrounding gel supports it from all sides, and afterward the network can be removed to leave a channel. This technique has achieved impressive results, like printing an entire lung-mimicking air sac network with delicate branches that would not survive in air but could be printed in a supportive fluid [105].

Finally, bioprinting with stem-cell-laden bioinks in situ in the operating room is being explored. This is essentially a 3D printing pen that a surgeon can use to deposit cells in a wound (a handheld extruder). While not as precisely controlled as a gantry system, it exemplifies how techniques may be hybridized with surgical techniques for direct tissue repair [121].

Each new method tends to be oriented toward overcoming a limitation of existing ones, whether it is achieving finer resolution, printing faster, handling more viscous inks, or better preserving cell function. As the field stands, extrusion, inkjet, and laser constitute the core, with others building upon them or combining them to widen the spectrum of what can be printed. Table 3 summarizes the commonly used 3D bioprinting techniques, highlighting their approximate achievable feature sizes, typical cell viability ranges, and representative application areas.

## 5. Structural and Mechanical Considerations

Bio-printed constructs must be engineered not only in terms of biology but also in their geometry and mechanical properties to serve their intended function. In many ways, this mirrors traditional scaffold-based tissue engineering considerations, with additional complexity because printing allows more intricate architectures. Key structural and mechanical factors include scaffold architecture (porosity, pore size, pattern), mechanical strength and stiffness, and biomechanical cues to cells, as well as how the construct may change mechanically over time (due to degradation or tissue maturation). Equally important are the rheological properties during printing and how they translate into the final microstructure (for example, layer adhesion, anisotropy) [125]. Below, we outline these considerations and best practices.

Most bio-printed tissues are printed with an internal lattice or porous architecture rather than solid, for two reasons: to allow nutrient diffusion and vascularization, and to achieve a target mechanical stiffness while using less material. The optimal pore sizes depend on the tissue: for bone tissue scaffolds, 200–500 µm pores are often cited to allow capillaries to grow in and osteoblasts to populate; for cartilage, a finer mesh might be used to mimic collagen fiber spacing. With 3D printing, one can design the porosity precisely. For instance, printing a grid with 700 µm strand spacing for bone, versus a tightly interwoven spaghetti-like filament pattern for cartilage, where high cell density is needed, and diffusion distances are smaller (because cartilage is avascular). The interconnectivity of pores is crucial: printed structures usually have fully interconnected channels by virtue of the layer-by-layer pattern, which is beneficial for fluid flow and cell migration (in contrast, some foaming or particulate-leaching scaffold fabrication methods can create closed pores that trap cells or fluids) [126,127]. Classical studies on scaffold porosity and optimal pore size provide useful guidelines for these design considerations [128].

The layer-wise nature of printing can introduce a slight anisotropy in structure. For example, a 0°/90° lattice printed layer by layer has pores that go straight through in the vertical direction (forming square channels), whereas a 0°/60°/120° repeating pattern might create a more complex polyhedral pore. These designs are chosen based on the needed perfusion vs. strength trade-off. A more porous scaffold gives better nutrient transport but is mechanically weaker. Fortunately, since design is flexible, one can incorporate features like gradients in porosity [129].

Bio-printed constructs, especially those intended for implantation, often need to have mechanical properties (like elastic modulus) in the ballpark of the target tissue to function properly and avoid stress shielding or collapse. For example, bone constructs should ideally exhibit elastic moduli in the tens to hundreds of MPa for cancellous bone (and several GPa for cortical bone), values that pure hydrogels cannot achieve [130]. Therefore, strategies such as printing a stiff thermoplastic framework or incorporating ceramic particles in the bioink are used to reinforce the construct. Even for soft tissues, matching the modulus is important. If a printed cartilage patch is too soft, it will not bear load in a joint. If too stiff, it may not integrate or could damage opposing cartilage [131].

Bioprinting allows control over filament alignment and lay-down pattern, which can tailor mechanical anisotropy. For instance, by printing all filaments aligned in one direction in a layer (instead of a grid), one can create a material that is stiffer along the filament direction than perpendicular, akin to fibrous soft tissues like tendons. Such anisotropic scaffolds have been explored for muscle and ligament tissue engineering. Another approach is printing a “sheath–core” composite filament (via coaxial nozzle) where the core is a stiff polymer and the sheath is a soft hydrogel with cells. This yields a filament that is strong yet bioactive. Mechanically, printed scaffolds often behave like cellular solids, and one can use modeling tools (like finite element analysis or analytical formulas for honeycombs) to predict how a given pattern will respond to stress. This is an advantage over random porous scaffolds, where such predictions are harder [132,133].

One must consider that the initial mechanical properties of the printed construct may change over time. As new extracellular matrix is deposited by cells, the tissue often stiffens (e.g., cells crosslink collagen and produce more of it, increasing modulus), but if the original scaffold material degrades, that could lead to softening. The ideal scenario is that initially the scaffold provides enough stiffness for handling and possibly implant load-bearing, then gradually transfers load to the developing tissue as it degrades (biomechanical matching). Many bio-printed cartilage patches, for example, start soft (to avoid hurting native tissue) and rely on the cells to produce matrix that eventually yields functional stiffness.

The layered nature of 3D printing can introduce a stepped surface topology (especially on curved surfaces of a print). In tissue engineering, this is generally not problematic; in fact, surface roughness can enhance cell attachment in some cases. However, if printing something like a bone-articulating surface, one might consider post-processing to smooth it or printing at a finer layer thickness for a smoother result. Interfaces between printed regions are critical. A printed implant must integrate mechanically with host tissue; designing interlocking or porous margins can help tissue interdigitate and form a strong bond. For example, a bio-printed bone graft might have an outer porous zone that encourages host bone ingrowth to anchor it.

Many bio-printed constructs use biodegradable materials (like alginate, fibrin, PLA, etc.), which gradually break down. The rate of degradation must suit the tissue’s healing/regeneration rate. If a scaffold with a mechanical role degrades too quickly, the new tissue may not yet be mature enough to handle loads, and the construct could fail. Conversely, if it degrades too slowly or not at all, it might hinder complete tissue regeneration or cause a chronic foreign body response. For instance, in a bio-printed cardiac patch, one might use a hydrogel that mostly degrades over a few months, by which time the hope is that the seeded cells have integrated with the heart and laid down their own matrix. Monitoring mechanical changes (using in vitro culture with mechanical testing at intervals) can be done to ensure that initially the patch is strong enough, and later it does not become a mechanical liability [125,127].

Cells respond to the stiffness of their substrate (mechanotransduction) and the presence of stress or strain. Therefore, the mechanical design of a bio-printed construct is not only about global properties. It is also about what the cells “feel”. For example, printing very thin filaments that each cell spans between may create a suspension-like environment, causing cells to elongate in a certain way. Or a stiff inclusion in an otherwise soft matrix can create local strain concentrations when the tissue is loaded. These effects can influence differentiation. Mesenchymal stem cells tend to become bone cells in stiffer surroundings and fat cells in softer ones, for instance. Bioprinting allows one to spatially vary stiffness by material patterning. This is a form of the 4D aspect. The structure causes biology to evolve differently in different zones. Furthermore, if a tissue will experience dynamic loading in vivo (like pulsatile blood pressure in a printed vessel, or compressive loading in printed cartilage), the construct should be designed accordingly. That might mean ensuring printed fibers are oriented to bear the expected loads, or that the bioreactor training in vitro applies those loads to condition the tissue [125].

In summary, just as CAD design is central to mechanical engineering, CAD design is central to bioprinting mechanical outcomes. By planning filament geometry, orientation, layer configurations, and material distribution, one can tune porosity, permeability, stiffness, and strength of the bio-printed object to meet specific requirements. Table 4 outlines guidelines for designing bio-printed scaffold architecture for various tissues. It is also crucial to remember that printed scaffolds must ultimately accommodate vascularization. Often, the mechanical design must leave enough space or channels for blood vessels to infiltrate, which can be as important as the mechanical integrity itself for the long-term viability of thick constructs.

When creating bioink and printing scaffolding, it is important to study the behavior of cells due to the print, so that design parameters can be modified and improved. Key parameters for scaffolding include geometry, density of material, pore size and porosity, mechanical stability, biocompatibility, and degradation.

Porosity and pore size are vitally important for cell migration, proliferation, and the exchange of nutrients. However, a large pore size could jeopardize the scaffold’s strength. Achieving a balance of pore size to scaffolding surface area can be done through pore gradient scaffolds, where the scaffold changes pore size based on the natural pore formation of the tissue, rather than having a uniform pore size over the whole surface area [140]. The geometry of the scaffold filament or infill pattern while printing directly affects mechanical strength and stability. Generally, the filaments are layered in a lattice pattern, such as honeycomb, rectangular, or zig zagged/diamond shaped. The primary use of scaffolding is to provide mechanical strength to the biomaterials, so it is important that the scaffolding maintains its shape after the printing process and while implemented in the host body, especially in load-bearing applications such as bone tissue. The elastic modulus of the scaffold should be similar in value to the tissue it is replicating for the best tissue healing and cell behavior [15]. Degradation of the scaffold should match tissue regeneration rates, so that the new cells produce at the same rate the scaffold dissolves to maintain structural integrity. If degradation of the scaffold occurs prematurely, the print is at risk of collapse [127]. If the degradation occurs too slowly, then the immune system of the host body is likely to respond, and the print region will become inflammatory. The material itself cannot be toxic or produce unsafe amounts of toxic byproducts during metabolism. This is especially the case for synthetic polymers, such as PLGA and PLA, which produce acid byproducts [125] and natural polymer, such as collagen and gelatin, which produce enzymatic byproducts [141].

## 6. Applications of 3D Bioprinting

3D bioprinting is being applied across a spectrum of biomedical fields. Here we highlight several major application areas and provide examples of how bioprinting is enabling new solutions:

### 6.1. Tissue Engineering and Regenerative Medicine

Bioprinting is perhaps most effective for its potential to create transplantable tissues and organs on demand. While whole complex organs are not yet available, many implantable tissue types have been bio-printed in simpler forms.

Bone and Cartilage: Bio-printed bone grafts typically involve a stiff scaffold (often printed with a polymer like PCL or with calcium phosphate cement) combined with osteogenic cells. For example, researchers have printed patient-specific bone substitutes by extruding a composite of hydroxyapatite particles in a biodegradable polymer matrix seeded with stem cells. These constructs, when implanted in animal bone defects, supported new bone formation and gradually degraded [142,143]. Cartilage, being simpler (consisting mainly of chondrocytes and ECM), has been bio-printed using cell-laden hydrogels like gelatin/alginate blends, often reinforced with a printed fiber framework to match the load-bearing requirements of joint cartilage. One noteworthy case is the bio-printed human ear mentioned earlier. Living auricular cartilage cells were printed in a collagen bioink into the shape of an ear and then matured. The implant developed cartilage stiffness and was successfully grafted in a human patient, retaining shape and integrating with surrounding tissues. This exemplifies how bioprinting can achieve the complex geometry of an external organ with the biological composition of native tissue [144,145].

Skin and Wound Healing: Skin is a multi-layer tissue with a simpler structure (mainly keratinocytes on top of fibroblasts, plus some appendages like hair follicles). Bioprinting has been used to fabricate skin patches wherein epidermal and dermal layers are printed sequentially. For instance, a bio-printed skin substitute might involve inkjet-printing human keratinocyte droplets onto a collagen gel containing fibroblasts to create a stratified structure [146]. This approach yields a skin graft with appropriate cell organization that can be directly applied to a wound. Studies in animal models have shown that bio-printed skin promotes faster healing and more normal skin architecture compared to cell suspensions or acellular grafts. Companies and research groups are actively developing skin bioprinters that could eventually be used in clinics to “print” skin directly onto burn wounds, as demonstrated in proof-of-concept with mice [147].

Vascular and Cardiac Tissues: Bioprinting small-diameter blood vessels or vascular networks is a critical steppingstone toward larger organs. Using coaxial extrusion, teams have bio-printed tubular structures made of human endothelial and smooth muscle cells in collagen/fibrin gels, achieving vessel-like constructs that can carry fluid. These printed vessels can be connected to bioreactors to perfuse them, and some have remained patent and matured into functional endothelium [148]. For cardiac tissue, bio-printed cardiac patches roughly a few centimeters in size have been made by printing cardiac muscle cells (derived from stem cells) in soft gels along with supporting cells and vasculature channels. One famous example is the mini heart from Tel Aviv University. Although it was too small to function as a transplant, it demonstrated the feasibility of printing cardiac cells with a patient’s own bioink (ECM from that patient) to form a unified construct [149]. More practically, cardiac patches containing organized cardiac fibers and vessel networks have been bio-printed and shown to integrate with host heart tissue in animal models, improving heart function after a myocardial infarction (heart attack) by contributing contractile cells and secreting growth factors to heal the injured heart [150].

Organs and Complex Constructs: Complete solid organs (kidney, liver, lung) are far more complex due to their size and functional intricacy. However, partial reconstructions and organ-like tissue blocks have been printed. For example, liver tissue lobules—small cylindrical units of liver structure—have been bio-printed with human hepatocytes and endothelial cells. These 3D liver tissues, only a few millimeters thick, are used in drug testing and have remained functional (detoxifying ammonia, producing albumin, etc.) for extended periods in vitro [151]. They hint that, in the future, assembling multiple printed lobules could form a larger liver graft. Researchers have also printed airway splints for pediatric tracheomalacia (a condition where the trachea collapses) using bioprinting of stiff polymers that are not cell-laden, but in some cases, designs incorporate a coating of bioink with cartilage cells to integrate with the native trachea. Kidney bioprinting has seen development in printing kidney proximal tubule models for drug testing. A true kidney would require addressing the challenge of millions of nephron units and their precise arrangement, which is an ongoing area of research with bioprinting of kidney organoids (mini-organs) as a first step [152,153].

In all these regenerative applications, a common theme is that bioprinting enables a level of personalization and complexity in the implant that traditional scaffold molding or manual cell seeding cannot easily achieve. For instance, an ear or jawbone with a very specific patient-specific geometry can be bio-printed to fit exactly, and with cells pre-positioned, potentially reducing the time needed for the tissue to populate and mature after implantation. Bioprinting can also incorporate conduits for nerves or vessels directly into the design of implants, something that conventional grafts cannot.

### 6.2. In Vitro Tissue and Disease Models

Another major application of bioprinting is creating advanced 3D cell culture models for research and pharmaceutical testing. These are often not intended for implantation, but rather to simulate human tissue physiology or pathology in the lab. By bioprinting human cells into organ-mimicking structures, scientists can get more predictive models for drug screening, disease progression, and personalized medicine:

Tumor Models: Bio-printed tumors allow studying cancer in a 3D context with multiple cell types (cancer cells, stromal cells, immune cells) arranged as they would be in a tumor. For example, a group might bio-print a small cube of tissue with breast cancer cells surrounded by fibroblasts and endothelial cells, creating a tumor microenvironment that can be perfused with nutrients. This model could then be used to test chemotherapy penetration or immunotherapy efficacy in conditions that resemble a real tumor better than 2D cultures or simple spheroids. Bio-printed tumor models have shown different drug responses compared to 2D, often more resistant (as real tumors are), thus providing a more stringent test bed for new drugs [154].

Organ-on-a-Chip Systems: Bioprinting can complement microfluidic organ chips by providing the 3D tissue that lives in the chip’s chambers. For instance, a liver-on-a-chip device could include a bio-printed mini-liver tissue containing hepatocytes and supporting sinusoidal cells, linked to a microfluidic channel that provides flow (simulating blood). Such a system can mimic the metabolism of drugs and their toxicity on the liver more realistically. A heart-on-a-chip might use a ring of bio-printed cardiac tissue that beats and can be subjected to mechanical load while measuring contractile force. Bio-printed renal (kidney) proximal tubule models have been made by printing renal epithelial cells in a tubular geometry inside a chip, recreating the reabsorption function of kidney tubules and allowing testing of drug-induced kidney injury [155,156,157].

Disease-Specific Models: Because bioprinting can use patient-derived cells (for example, from biopsies or induced pluripotent stem cells), it is possible to create patient-specific disease models. A compelling case is bioprinting tissue from a patient with a genetic disease to test how that tissue responds to various treatments in vitro, effectively a personalized “twin” of the patient’s tissue. Similarly, bio-printed cardiac tissues from a patient who had a particular heart disease could be used to test drugs for efficacy and safety, specifically on that patient’s cells [158].

Physiological Models: Bioprinting also enables models to study basic human biology, such as printing model placental tissue to study nutrient exchange from mother to fetus, or printing neuronal networks for brain circuit research. A recent example involved bioprinting a small piece of neural tissue with neurons and supportive glial cells in a layered structure to test electrophysiological development and screen neurotoxic chemicals [159]. Another example is using bioprinting to arrange pancreatic islet cells in a 3D configuration reflecting the islets of Langerhans, to better understand diabetes in a controlled setting [157,160].

These in vitro applications do not face the same vascularization challenge as implants, because they can be perfused externally or are small enough to rely on diffusion. Bioprinting’s advantage here is reproducibility and complexity (embedding multiple cell types, creating relevant micro-architectures like villi, crypts, or alveoli shapes). The pharmaceutical industry is keen on such models, as early drug testing on bio-printed human tissues could reveal toxicity or metabolism issues that might not be evident in animal models, thus derisking drug development. Regulatory agencies are also warming up data from advanced tissue models as part of Investigational New Drug (IND) filings [161].

### 6.3. Personalized Implants and Prosthetics

Beyond growing biological tissues, bioprinting intersects with the field of medical devices by enabling customized implantable structures that incorporate biological elements. Some examples include:

Hybrid Prosthetics: Bioprinting can produce interfaces between synthetic prosthetic parts and the body. For instance, consider a titanium orthopedic implant (like a hip joint). With bioprinting, one could fabricate a layer of bone-like material with the patient’s bone cells on the surface of the metal implant, encouraging faster integration with the patient’s bone. There are experimental approaches where porous metal implants are seeded with bio-printed bone marrow stem cells in a gelatin matrix to create a living coating on the implant that then mineralizes in vivo [162].

Dental and Craniofacial Implants: The dental field is exploring bio-printed gums, periodontal ligament, or even tooth structures. While printing an entire tooth is extremely challenging (due to enamel hardness and such), researchers have bio-printed tooth root analogs with stem cells that were able to initiate dentin formation [163]. More near-term, bio-printed bone or gum tissue can be used in jaw reconstructions for dental implants. For example, after tumor removal in the jaw, a bio-printed piece of bone with the correct shape and a tooth socket could be implanted, containing cells that will generate new bone and integrate with the remaining jawbone, as well as perhaps periodontal ligament cells to eventually attach to an artificial crown [164].

Orthopedic and Spinal Implants: Bioprinting has been used to create intervertebral disk substitutes containing both cartilage-like and fibrocartilage regions, aiming to replace degenerated disks in the spine [165]. These implants can be made patient-specific in size and curvature, and initial studies in small animals show they can maintain disk height and facilitate tissue regeneration between vertebrae. Bio-printed constructs for tendon or ligament repair (e.g., an ACL graft) are being explored, where the gradient from tendon to bone can be fabricated in one continuous print (soft collagen at one end, stiff mineralized material at the other) [166].

Organ Patches and Decellularized Scaffolds: Another concept is bioprinting cells onto decellularized organ scaffolds [167]. For example, a whole liver or kidney from a donor can be decellularized to leave a collagen matrix scaffold. Instead of relying on random cell seeding, bioprinting can be used to placement-control the re-population of that scaffold by printing hepatocytes into the liver lobule regions and endothelial cells along the vessel channels. While not freeform printing (the scaffold acts as a guide), this combination of decellularized matrices (which retain the original organ architecture) and bioprinting is a promising path to create transplantable organs in the longer term [168].

### 6.4. Spheroid-Based Bioprinting

Spheroid-based bioprinting has emerged as a versatile approach for constructing complex, scaffold-free tissue constructs by leveraging the inherent self-assembly properties of cell aggregates. In this strategy, cells are first organized into spheroids, which are three-dimensional multicellular clusters that can then be precisely positioned to form larger, functional tissue architectures. Norotte et al. [169] pioneered this concept by demonstrating scaffold-free vascular constructs formed from cellular spheroids, establishing a foundation for vascular tissue engineering without the need for supporting biomaterials.

Subsequent studies have extended these principles to heterogeneous, vascularized tissues. Kolesky et al. [170] successfully fabricated complex, multi-cellular constructs with embedded vascular networks, illustrating the potential for generating clinically relevant tissue geometries. Marga et al. [171] introduced organ-module and aggregate-based assembly strategies, highlighting the modular nature of spheroid fusion for organ-scale tissue fabrication. Recent advances have further improved the precision and viability of spheroid placement. Minaeva et al. [172] demonstrated laser-assisted bioprinting as a gentle yet highly accurate technique for depositing spheroids, minimizing mechanical stress while enabling high-resolution tissue patterning. Complementing these technological developments, Jakab et al. [173] provided a comprehensive overview of self-assembly principles, emphasizing the biological mechanisms that drive spheroid fusion and tissue maturation. Practical methodologies for spheroid-based bioprinting, including considerations of spheroid size, density, and culture conditions, were systematically outlined by Daly et al. [174], providing essential guidance for reproducible construct fabrication.

Overall, spheroid-based bioprinting combines the advantages of scaffold-free tissue assembly with high architectural fidelity, making it a promising approach for engineering functional tissues and organ modules. Its integration with vascularization strategies and laser-assisted techniques positions it as a critical method in next-generation tissue engineering applications.

### 6.5. High-Throughput Testing and Pharmacology

Bio-printed tissues are also enhancing high-throughput screening processes. Companies have 3D-bioprinted arrays of mini tissues (e.g., 96 little liver tissues on one plate) to test drug toxicity on all of them systematically. This yields more reliable data on how a drug might cause liver injury or affect heart electrical activity than 2D cell monolayers. In toxicology, regulatory agencies are interested in replacing some animal tests with human-cell-based assays. For example, testing cosmetic ingredients on bio-printed skin models, or environmental toxin effects on bio-printed lung microtissues [175].

### 6.6. Educational and Surgical Planning Models

Although they do not always contain living cells, bio-printed anatomical models can be very helpful for surgical training and planning. By printing a model of a patient’s organ, surgeons can practice complex surgeries beforehand [176]. For instance, a patient-specific kidney tumor model could be bio-printed with a hydrogel that mimics kidney parenchyma and colored regions for the tumor and blood vessels. Surgeons can rehearse the excision and see if their approach will likely spare the important vessels. Some of these models incorporate animal blood or blood-mimicking fluids to simulate bleeding. While one might not need “bioprinting” per se for making a model (regular 3D printing might suffice with silicone or polymers), some groups use bioprinting to get gelatin-based models that feel more like real organ tissue. These models greatly aid in education; medical students can dissect a bio-printed organ model that has correct anatomy, or practice suturing on a bio-printed skin pad.

In summary, the applications of 3D bioprinting are diverse, ranging from building tissues for therapy to creating model systems for science and industry. Not every application demands the highest resolution or the most complex multi-material printer. The needs differ. For producing a large bone implant, what matters is mechanical strength and biocompatibility of materials; for producing a neurodegenerative disease model, what matters is cell type arrangement and microenvironment cues. Bioprinting, with its flexibility in design and composition, is uniquely positioned to meet these varied demands, bridging engineering and biology to address both medical and research challenges.

## 7. Challenges and Limitations

Despite the remarkable progress in 3D bioprinting, significant challenges remain before the full vision (such as printing whole transplantable organs) can be realized and before current bio-printed products become routine clinical options. Key challenges include vascularization of thick tissues, maturation and long-term functionality, scale-up and manufacturing consistency, regulatory hurdles, and ethical considerations.

### 7.1. Vascularization and Tissue Thickness

Perhaps the greatest biological challenge is how to provide nutrients and oxygen to cells in large bio-printed constructs. In native tissues, networks of blood vessels (down to capillaries ~5–10 µm in diameter) permeate the tissue, ensuring no cell is more than ~100–200 µm from a capillary. In bioprinting, when we create a large tissue (more than a millimeter or two thick), the embedded cells in the interior will quickly die if we do not establish some perfusion [177].

Current bioprinting methods have partial solutions. One can print larger channels or conduits (hundreds of microns) and then endothelialize them to serve as larger blood vessels, but the challenge is getting down to the micro-capillary scale and having those self-assemble or develop after implantation [178]. Another strategy is to include angiogenic factors (like VEGF) in the bioink to promote ingrowth of host vessels once implanted [179]. Another approach is co-printing supporting cells (like stromal cells) that facilitate capillary formation, thereby essentially encouraging the tissue to vascularize over time. Even so, ensuring that a printed thick organ will uniformly vascularize is unresolved. This challenge also affects in vitro tissues. Bioreactors with perfusion can help, but diffusion limits remain. Therefore, researchers are exploring the use of pre-vascularized bioinks. While some success has been seen in animals, full hierarchical vascular networks with arteries, arterioles, capillaries, venules, and veins all integrated is still a frontier [180]. These strategies and challenges have been extensively reviewed in the vascular tissue engineering literature, highlighting both the biological and engineering considerations for promoting functional vascularization in engineered constructs [181,182,183].

### 7.2. Maturation and Functional Integration

Printing the correct cells in the correct place is often just the beginning. Those cells then need to mature and organize into a functional tissue. In many cases, bio-printed tissues initially resemble an immature version of the target. For example, a bio-printed cardiac patch may have heart muscle cells present, but they might not beat in unison strongly until they electrically couple and align over some weeks of culture—akin to how fetal heart cells mature after birth. Achieving this maturation may require biochemical cues (growth factors) and biophysical cues (electrical pacing or mechanical stimuli) provided in a bioreactor [184]. Even then, some cell types, like induced pluripotent stem cell-derived tissues, may not fully mature to adult functionality in a lab setting. When a construct is implanted, one hopes that the native environment will encourage further maturation, but it is not guaranteed. Another aspect is integration with the host tissue. A bio-printed tissue will need to hook up with host blood vessels, nerves, and mechanical integration. Achieving seamless integration remains challenging. There is a risk that the bio-printed tissue will not sufficiently connect and will essentially be an active “patch” but not fully part of the organ’s coordinated function [185].

### 7.3. Stability and Biodegradation Control

Many bio-ink materials are chosen for good initial printability and biocompatibility, but their behavior in vivo over longer time scales is less understood. Some hydrogels may swell excessively or degrade too quickly once implanted. There is a fine balance where a scaffold should degrade at a pace so that the newly formed tissue can take over load-bearing. If the degradation products are acidic or cause inflammation (like PLA’s lactic acid), they can harm the very tissue being regenerated [36]. Thus, tuning degradation is a challenge that requires further material innovation. Similarly, ensuring long-term stability of the printed structure’s shape is important. There is often remodeling by cells (cells can contract collagen gels, for instance, shrinking the construct). A printed shape might warp or shrink in size during maturation in unpredictable ways if not carefully monitored or mechanically constrained. This is an area of active research, looking at how to design bioinks and scaffold architectures that maintain intended geometry through tissue growth and remodeling [186].

### 7.4. Scaling up Production and Throughput

While printing one or a handful of constructs in a research lab is feasible, scaling up to manufacturing dozens or hundreds of tissue constructs per day (which might be needed for commercial therapy or for large-scale drug screening) presents logistic and technical challenges. Bioprinting can be slow for large volumes, and keeping a sterile production pipeline with living cells is non-trivial. Bioprinter hardware would need to be robust and possibly automated with minimal human intervention to ensure consistency. There is also the challenge of cell sourcing, making a large tissue requires billions of cells. For patient-specific tissues, you need to expand the patient’s cells or differentiate stem cells in sufficient quantity, which can take time and resources. If using allogeneic cell lines (cells from a universal source), immunocompatibility needs to be addressed [187].

Standardization is not yet established. Different labs use different bioink recipes even for the same application, and this variability can lead to different outcomes. To move toward clinical or industrial use, more standardized “bio-fabrication protocols” will be needed so results are reproducible across batches and sites. Additionally, current bioprinting often has a high rate of trial-and-error, and yields might be low initially. Improving this yield is essential for economic production [188].

### 7.5. Regulatory and Ethical Barriers

Bio-printed products blur the lines between medical devices, biologic products, and combination products. Regulatory bodies like the FDA are still determining the best frameworks to evaluate these. Key concerns include ensuring safety (e.g., no harmful immune or tumorigenic potential), efficacy, and quality control. For instance, a bio-printed organ might have slightly different cell distributions each time, and it has to be decided on how to define and measure an acceptable range. Traditional pharmaceuticals have very strict batch release criteria via chemical analysis. For a living product, one might need advanced imaging or biomarker assays to verify that each printed tissue meets specifications. Regulatory guidance documents from the FDA and EMA provide initial frameworks, but clear standards for living, patient-specific bio-printed products are still evolving [189]. Another regulatory issue is the long-term follow-up. If you implant a bio-printed tissue, how long do you need to monitor patients for possible adverse outcomes? This increases the burden of proof for approval [190].

Ethically, if bioprinting uses a patient’s own cells, it raises fewer ethical issues, as they are autologous and personalized. But if the field moves toward using, say, embryonic stem cells or modified cells to create universal bioinks, there could be ethical debates around sources of cells (embryonic or fetal tissues) and genetic engineering. Another issue is equity of access. These therapies could be very expensive initially; ensuring they do not widen healthcare disparities will be a challenge. There is also an interesting debate on “enhancement”. Could bioprinting be used not just to replace lost function but to enhance humans? Society and regulators would need to draw lines on acceptable uses [191].

### 7.6. Technical Complexity and Multidisciplinarity

Bioprinting inherently requires expertise in cell biology, materials science, mechanical engineering, software, surgery, etc. Developing a successful product means bridging all these fields, which is challenging in itself. A lot can go wrong. Cells may differentiate incorrectly, materials may not print as expected, etc. As such, bioprinting projects often have high research and development overhead. For widespread adoption, the processes need to be simplified and robust. Currently, a typical bioprinting experiment might involve many manual steps (cell harvest, encapsulation, printer calibration, etc.), each of which can introduce variability. Automation and closed-loop controls (sensors checking printing fidelity in real-time) are in early development, which would improve reliability [192].

Quality control of intermediate and final products is another challenge. This involves the non-destructive test of a living product. Techniques like live/dead staining are not feasible for an implant that will be delivered. Advanced imaging (like optical coherence tomography, ultrasound, or even MRI) might be employed to inspect print quality and cell distribution before release. For implanted tissues, safety concerns include ensuring no residual contaminants [193].

One particular challenge in implantation is the host’s immune reaction. Even “autologous” constructs can provoke immune responses because the process of manufacturing could introduce subtle changes (e.g., different gene expression due to cell expansion on plastic). Foreign biomaterials, unless fully degraded, may cause chronic inflammation or fibrosis (scar tissue encapsulation). For bio-printed organs containing allogeneic cells, immunosuppression would likely be needed, similarly to organ transplants. There is research into making “universal cell lines” that lack certain cell-surface markers to evade immune detection, which could benefit bioprinting, but that introduces additional layers of genetic modification and safety evaluation [194].

### 7.7. Commercial and Logistical Challenges

The path to clinical use will also need demonstration of cost-effectiveness and logistical feasibility. The production of bio-printed tissues may require specialized facilities, such as GMP cleanrooms. For hospitals, adopting bioprinting might mean maintaining a facility akin to a cell therapy lab, which not all hospitals can do. Alternatively, centralized facilities might produce the tissues and ship them, which raises issues of preserving viability during shipment. Keeping tissues alive and ready for surgery at the right time requires coordination [195].

## 8. Future Outlook

The future of 3D bioprinting is very promising, and numerous trends are emerging that aim to address current limitations and open new possibilities. Several key areas are expected to see significant advancement:

### 8.1. Integration of AI and Machine Learning

Artificial intelligence (AI) and machine learning (ML) are poised to play a big role in optimizing bioprinting. AI can help in design automation; given a desired tissue function, algorithms could propose an optimal spatial distribution of cell types and materials. Machine learning can also analyze vast amounts of experimental data to find patterns (e.g., which bioink compositions yield the highest viability for a given cell, or what printing speed and pressure minimize clogging). We may see “smart bioprinters” that have embedded sensors (cameras, pressure monitors) feeding data to an AI system in real-time; the system could adjust parameters on the fly to correct any deviations, essentially creating a self-optimizing print process. AI could also assist in quality control by learning to recognize from images if a printed tissue is likely to develop problems (for example, an undesired pore or a region of low cell density). On the design side, generative algorithms might be used to generate scaffold patterns that maximize certain mechanical or biological performance metrics that a human designer might not conceive of easily. Additionally, patient-specific aspects (like using patient imaging) can be combined with AI to plan complex prints—e.g., designing a vasculature layout that would best anastomose with a particular patient’s existing vessels [196,197].

### 8.2. Advanced Materials and Bioinks

The next generation of bioinks will likely include smart materials that respond to stimuli or that can encourage cells to behave in certain ways. For example, bioinks that respond to magnetic fields (embedding magnetic nanoparticles) could allow external magnets to remotely guide the positioning of cells or the alignment of fibers during or after printing. Bioinks may be developed to release signals over time in a controlled manner, e.g., tiny polymer microspheres in the ink that slowly release growth factors like BMP-2 for bone or VEGF for blood vessels, giving the printed tissue sequential “instructions” over weeks (first encourage blood vessel growth, then encourage tissue-specific matrix deposition) [198]. Self-healing hydrogels are another interesting area. These are materials that can bond back together if a minor rupture occurs. This could help printed tissues remain intact under stress or if inadvertently cut during surgery [199].

We will also see multi-material bioprinting taken further, not just two or three inks but potentially dozens of different cell and material “inks” in one construct, truly mimicking the complexity of an organ (which can have dozens of cell types). To manage that, printers with many printheads or very clever ways of switching inks rapidly will be developed [200].

Another frontier is bioprinting at the nano- or microscale level. Techniques like two-photon polymerization can already create sub-micron features, and combining them with biocompatible materials and seeding cells might enable printing of basal lamina structures or capillary networks with tiny diameters. Though printing cells directly at sub-micron resolution is unrealistic (cells are 10 µm), printing acellular guiding structures that cells can later populate (like nano-grooved surfaces to align cells) can be done [201].

### 8.3. Bioprinting with Microorganisms

An emerging direction in biofabrication involves the bioprinting of microorganisms, expanding the scope of living material engineering beyond mammalian cells. Microbial bioprinting enables the creation of functional architectures for engineered living materials, biosensors, microbiome-on-chip platforms, and bioactive tissue scaffolds. Laser-based approaches have allowed high-resolution placement of bacteria and other microbes. This provides precise spatial control while maintaining viability [202,203]. Complementary strategies using bacteria-laden bioinks further facilitate the development of three-dimensional microbial communities with tailored functionality [204].

Recent advances in microbial bioprinting have moved beyond proof-of-concept demonstrations toward precision-controlled deposition and functional microbial patterning. In extrusion-based systems, printing resolution typically ranges from 100 to 300 µm depending on nozzle diameter and bioink rheology. In contrast, inkjet-based systems can achieve droplet volumes in the pico- to nanoliter range with deposition frequencies exceeding 1 kHz. Reported post-print microbial viability commonly exceeds 80–95%, provided shear stress and pressure conditions are optimized [205,206]. LAB, particularly LIFT-based approaches, enables nozzle-free deposition of microorganisms with spatial resolution below 50 µm and minimal mechanical stress. The absence of shear-inducing nozzles reduces clogging and enables precise patterning of bacterial and yeast populations, facilitating high-density microbial arrays for biosensing, metabolic engineering, and synthetic biology applications [207].

Beyond conventional bioprinting, an emerging research direction is the laser engineering of microbial systems. With this, the tightly focused laser pulses are used to isolate, manipulate, or selectively transfer individual microbial cells. This approach enables high-resolution patterning, the isolation of rare microorganisms, and the targeted deposition of specific strains from heterogeneous populations. Such capability is particularly valuable for the discovery of novel antibiotic-producing microorganisms and biologically active compounds. Laser-based microbial isolation methods enable spatially selective extraction of viable cells from complex environments. This reduces contamination risks and improves cultivation efficiency. Compared to traditional plating or microfluidic sorting, laser-mediated techniques provide micrometer-scale precision and rapid single-cell manipulation without extensive preprocessing steps [208,209].

While microbial bioprinting offers exciting opportunities, it also introduces unique challenges, including stricter biosafety considerations, specialized process control, and differences in growth and metabolic behavior compared to mammalian cells [210]. Incorporating microbial systems into biofabrication workflows thus represents both a promising expansion of bioprinting capabilities and a distinct set of technical considerations that must be addressed.

### 8.4. 4D Bioprinting and Dynamic Tissues

The concept of 4D printing (time being the fourth dimension) will likely be heavily explored. This involves printing constructs that change shape or properties in response to physiological conditions or as they mature. For instance, a tissue could be printed in a compact form for surgical implantation through a small incision, then once inside the body, it unfolds or expands to a larger size (perhaps triggered by temperature or hydration) [211]. Or a printed scaffold might gradually stiffen as cells produce matrix, due to secondary crosslinking mechanisms that activate after a delay. We can also envision tissues that have “on-demand” functionality—e.g., a bio-printed endocrine patch that releases insulin when glucose levels rise (some scaffolds contain cells engineered to sense glucose), effectively acting as a smart device.

Beyond shape-morphing and time-dependent functionality, current research in 4D bioprinting increasingly focuses on programmable cell–material interactions, where bioinks are designed to dynamically modulate stiffness, degradation rate, or ligand presentation in response to cellular activity or external stimuli [212]. Such systems enable constructs to evolve alongside tissue maturation, better recapitulating developmental and regenerative processes in vivo.

In parallel, the emerging concept of 5D bioprinting extends these ideas by incorporating multi-axis deposition and curvature-aware printing to fabricate mechanically robust, anisotropic tissues with complex geometries, such as load-bearing cartilage, vascular grafts, or musculoskeletal interfaces. When combined with smart, stimuli-responsive materials, 5D approaches offer a pathway toward dynamically adaptive, structurally optimized living systems that respond to both biological and mechanical cues over time [213].

### 8.5. Bioprinting Combined with Electronics or Sensors

Future bio-printed organs might integrate bioelectronics. For example, printing cardiac tissue with embedded flexible electrodes or printed electronics to monitor function or stimulate the tissue [214]. This combination of tissue and technology could lead to “bionic” constructs that not only replace tissue but also provide real-time feedback or therapeutic intervention (like a printed pancreas that not only has islet cells but also glucose sensors and a built-in release mechanism for insulin). Already, people are looking at printing conductive materials (like graphene or gold nanowires) alongside cells to create tissues that can conduct electricity (good for the heart and nerves) [215]. There is also the possibility of incorporating drug delivery reservoirs in a printed implant, which can locally release an immunosuppressant drug to protect itself from rejection in the early phase, then stop releasing it once the danger period is over [216].

### 8.6. Standardization and Bio-Fabrication Ecosystem

As the field matures, we expect to see an ecosystem of supporting technologies: standardized cell lines for printing (much like standardized cell lines for manufacturing biologics), certified bioinks available off the shelf for certain applications, and software solutions for tissue design (perhaps an app where a clinician can input some parameters and get a printable design, rather than needing an engineer to draw it from scratch). Initiatives to establish bioprinting standards, such as defined testing methods for mechanical properties, cell viability assays specific to printed constructs, etc., are underway and will facilitate regulatory approval [217,218].

### 8.7. Regulatory Evolution and First Clinical Trials

Soon, we expect to see the first clinical trials of bio-printed tissues. Likely candidates are relatively small, simple tissues that address unmet needs: for instance, clinical trials are already planned (or just starting) for bio-printed cartilage in the knee, or bio-printed skin for burn patients, as well as bio-printed bone patches for cranial defects. Success in those will build confidence and pave the way for more complex internal organ trials, such as patching a heart or parts of a liver. Regulators will become more comfortable as more data emerges; they may also issue guidelines specific to bioprinting, which can actually help developers by clarifying what is required [219].

### 8.8. Ethical and Accessibility Advances

On the ethical front, with more progress, there will be efforts to ensure bioprinting is used responsibly, likely establishing frameworks for compassionate use (e.g., allowing a patient with no other options to try a bio-printed organ under experimental protocol). Technology might also be used in veterinary medicine as a stepping stone (e.g., bioprinting tissues for pets or endangered animals), which raises fewer ethical concerns against the concept and can gather useful data. Efforts to reduce cost will continue using more common materials, simplifying processes, and perhaps leveraging economies of scale in cell production (like centralized cell farms to supply many prints). Over time, as patents expire and techniques become widespread, we might even see open-source bioprinting recipes that enable broader [220].

### 8.9. Bio-Fabrication Service Bureaus

In the long run, one can imagine a hospital not necessarily owning a bioprinter, but rather sending patient data to a bio-fabrication center that prints the needed tissue and ships it sterilely the next day. Those service bureaus might handle multiple tissue types and would have specialized quality systems, making regenerative medicine more like ordering a personalized medical device. This would require robust shipping methods (maybe cold-chain or bioreactor-equipped shipping containers to keep tissues alive) [221].

### 8.10. Convergence with Other Technologies

Bioprinting will likely converge with gene editing, so that one can print tissues with cells that have been modified to avoid disease or enhance therapeutic factor secretion. It may also converge with CRISPR-based cell therapies by structuring those therapies in 3D [222]. Another interplay is with immunotherapy, e.g., printing a tumor model with a patient’s cancer and immune cells to test immunotherapy drugs on it (personalized tumor-on-chip), a near-term helpful application [223].

Another futuristic idea is in situ bioprinting, i.e., printing directly inside the patient during surgery (or even via minimally invasive approaches). There have been prototypes of printing devices mounted on surgical arms that could deposit cells onto an internal organ surface. In the future, a surgeon might use a robotic bioprinter to “fill in” a wound or resected area with new tissue on the spot, tailoring it in real time as needed. This is a bit far out, but not inconceivable, as it merges surgical robotics and bioprinting [224].

In summary, the future of 3D bioprinting points toward more complex, functional, and clinically oriented bio-printed constructs, aided by smarter technology and deeper biological integration. We expect to see incremental progress (improved viability, small in-human trials) in the short term, and possibly breakthrough applications like functional organ patches and large-scale tissue production in the longer term.

## 9. Conclusions

Three-dimensional bioprinting has evolved into a powerful bio-fabrication strategy with the potential to transform tissue engineering, drug development, and regenerative medicine. Precise spatial control over cells and biomaterials enables the fabrication of constructs that closely replicate native tissue architecture and function. Major bioprinting approaches, including inkjet, extrusion, and laser-assisted, offer distinct trade-offs between resolution, throughput, and cell viability. A diverse range of bioinks, including natural, synthetic, and composite materials, supports both biological functionality and mechanical stability. Scaffold design parameters such as pore geometry, layer orientation, and anisotropy play a critical role in governing mechanical performance and mass transport, requiring optimization for specific tissue applications. Advances in bioprinting have already enabled the fabrication of bone, cartilage, skin, vascular networks, and complex organ models, with demonstrated successes in cardiac repair, patient-specific cartilage reconstruction, and physiologically relevant in vitro models for drug screening. Together, these developments underscore the growing impact of bioprinting as a foundational technology for biomedical research and future clinical translation.

Yet, significant challenges remain. Key among them is the issue of vascularization, ensuring that printed tissues can develop or be supplied with microvasculature so that thick, clinically sized tissues survive post-implantation. Without solving this, organ-scale constructs will not progress. Bioprinting researchers are actively pursuing solutions such as sacrificial printing of vessel networks and inclusion of angiogenic factors. Maturation of printed tissues is another hurdle. Printing places the cells, but guiding them to form a functional tissue is equally critical. Advances in bioreactors, as well as bioprinting of supporting cell types (like printing nerve conduits to promote innervation), are likely to improve the functional integration of printed grafts.

Manufacturing scalability and regulatory considerations remain critical challenges for the clinical translation of bio-printed constructs. Bioprinting is a complex manufacturing process that must achieve high consistency and meet clinical-grade standards (GMP). Automation, AI-driven control, and rigorous standardization will be needed to move bioprinting from artisanal production into a reliable industry. On the regulatory front, initial products will likely be small and relatively simple (such as bio-printed skin or cartilage) to demonstrate safety before more complex organs are attempted. Ethical considerations, such as equitable access and appropriate use, will require attention as technology approaches clinical reality.

Despite these challenges, the trajectory of progress is very encouraging. The field of bioprinting has benefitted from the convergence of tissue engineering, stem cell science, advances in biomaterials, and 3D printing technology itself. Continued interdisciplinary collaboration will be essential. It is conceivable that, in the next decade, we will witness the first clinical trials of bio-printed tissues like personalized bone or cartilage implants, and perhaps the regulatory approval of bio-printed skin for treating wounds. Looking further ahead, each success with relatively simple tissues will build the foundation (in terms of technology, regulatory pathway, and clinical confidence) for tackling more complex organs. If a bio-fabricated kidney or liver still appears to be a distant goal, the steady improvements in vascularization strategies and the possibility of integrating bioprinting with techniques like organ decellularization (using printed cells to repopulate natural scaffolds) suggest it is not a question of if, but when such organs will become available.

Overall, 3D bioprinting is gradually transforming from a futuristic concept into a practical bio-manufacturing tool. Its ability to personalize treatments (by using a patient’s own cells and matching anatomical shapes) aligns perfectly with the trend towards personalized medicine, potentially reducing issues of graft rejection and improving repair of complex defects. Moreover, bioprinting’s contributions to creating realistic human tissue models in vitro are accelerating drug discovery and reducing reliance on animal testing, which has broad societal benefits. While significant work remains to overcome current limitations, the progress to date and the rapid growth of the bioprinting community justify optimism. With continued innovation and careful validation, it is foreseeable that in the coming years, bio-printed constructs will move from the benchtop to the bedside, providing new treatments for injuries and diseases that are difficult to address with traditional methods. The vision of on-demand printing of organs and tissues, once a science fiction trope, is steadily becoming a scientifically grounded pursuit, promising to usher in a new era of regenerative medicine where organ shortages and untreatable tissue damage become problems of the past.

## Figures and Tables

**Table 1:** Summary of Common Bioinks, Polymer Classification, Solubility, Crosslinking Method, Advantages, and Disadvantages.

Cell Ink	Solubility	Crosslinking Method	Advantages	Disadvantages	Applications	References
Agarose	Water-Soluble/Thermo-reversible gelation	Thermal Gelation	- Good shape fidelity, stiffness - Biocompatible - Reversible crosslinking	- Dispensing at high temperatures - Poor cell adhesion - High water retention	- Bone and Cartilage - Muscle - Drug Delivery	[41]
Alginate	Water Soluble	Ionic	- Fast gelation Biocompatible(Similar ECM to human tissue) - Tunable viscosity	- Hydrophilic nature causes poor cell adhesion - Limited bioactivity	- Bone - Cartilage - Cardiovascular - Nerve	[44,58]
Collagen	Soluble in low acidic aqueous solutions	Thermal or pH-induced self-assembly	- Native ECM Promotes cell adhesion, differentiation - Low immunogenicity	- Low viscosity - Print variability - Slow gelation - Poor mechanical strength	- Muscle - Cartilage - Cardiovascular - Bone	[59,60,61]
Gelatin	Water Soluble	Thermal or enzymatic can be photo crosslinked as GelMA	- ECM Bioactive - Supports cell proliferation - Modifiable	- Thermally unstable - Limited mechanical integrity	- Muscle - Cartilage	[54,62]
Chitosan	Soluble in and acidic solution of pH 6.3–6.5 or lower	Ionic, chemical	- Antibacterial - Biocompatible, Biodegradable	- Weak mechanical properties - Slow gelation rate	- Drug Delivery - Cartilage - Blood Vessels	[46,63]
Cellulose	Cellulose derivatives used are water soluble	Ionic, chemical agents	- Enhances viscosity, elasticity, porosity - Biocompatible - Strong mechanical support	- Limited cell adhesion - Poor biodegradability	- Bone - Cartilage - Wounds	[49,64]
dECM	Derivatives are water-Dispersible	Thermal, Enzymatic	Provides native biochemical cues, Promotes cell growth and differentiation	Variability, Complex preparation, Limited mechanical stability	- Bone - Liver - Kidney	[44,65,66]
Polycaprolactone (PCL)	Hydrophobic	Chemical, Photo	- Excellent mechanical strength - Excellent print fidelity - Modifiable degradation rate	- Lacks bioactivity - Poor cell adhesion	- Bone - Cartilage - Wound	[67,68]
Polylactic acid (PLA)	Hydrophobic	Photo, Chemical	- Biocompatible - Tunable degradation	- May produce acidic byproducts - Low degradation rate	- Bone - Cartilage - Liver - Drug Delivery	[69,70]
Polylactic-co-glycolic acid (PLGA)	Hydrolysis	Chemical, Thermal	- Slow and Controlled degradation - FDA-approved	- Acidic degradation products - Limited cell adhesion	- Bone - Cartilage - Liver - Drug Delivery	[71,72,73]
Polyethylene glycol (PEG)	Water Soluble	Chemical, Ionic	- Highly tunable - High permeability - Tunable degradation rate - Bioinert	- Lacks bioactivity - Poor degradability	- Bone - Cartilage - Drug Delivery	[74,75]
Pluronic	Water Soluble	Enzymatic, Photo	- Excellent print fidelity - Shear-thinning properties - Thermosensitive	- Lacks bioactivity - High degradation rate - Dissolves easily in aqueous media	- Sacrificial Scaffolding - Drug Delivery - Wound Application	[76,77]

**Table 2:** Types of Fillers, Functions, and Applications.

Filler Type	Examples	Main Functions	Usage in Bioprinting	Reference
Ceramic fillers (inorganic)	Hydroxyapatite, β-TCP, bioactive glass	Add rigidity; osteoconductivity (promote bone-like mineralization)	Bone and cartilage tissue scaffolds	[69,81]
Carbon-based fillers (inorganic)	Graphene oxide, carbon nanotubes	Improve mechanical strength; introduce electrical conductivity	Neural and cardiac tissues (electrically excitable), reinforcement of soft hydrogels	[82,83]
Metallic fillers (inorganic)	Gold nanoparticles, Zinc or Titanium oxides	Conductivity; antimicrobial properties (ZnO, Ag); X-ray opacity	Engineered cardiac patches (gold nanostructures for conductivity), wound dressings (Ag/ZnO for antibacterial effect)	[84,85]
Natural polymer fillers (organic)	Nanocellulose, chitin whiskers	Reinforce hydrogel network; improve viscosity and print fidelity	Cartilage and skin bioinks (to enhance mechanical stability while remaining biocompatible)	[86,87]
Synthetic polymer fillers (organic)	PCL fibers, PLGA microspheres	Provide structural support; controlled degradation releasing growth factors	Hybrid scaffolds for bone or muscle (printed alongside cells to bear load initially)	[73,88]
Nanoclays & silicates (inorganic)	Laponite (nanosilicate), bioactive glass particles	Rheology modifier (shear-thinning); bioactive ion release	Any bioink to improve printing consistency and cell differentiation (e.g., nanosilicates induce osteogenesis)	[89,90,91]

**Table 3:** Comparison of common 3D bioprinting techniques (approximate achievable feature size, typical cell viability, and example applications).

Bioprinting Method	Approx. Resolution	Post-Print Cell Viability	Typical Applications	References
Extrusion-Based	200–1000 μm	50–80%	Large tissue constructs (bone and cartilage scaffolds, organ models)	[106,99]
Continuous inkjet	50–300 μm	80–95%	Biomolecule patterning on substrates, printing long filaments of acellular bioinks	[122]
E-jet (electrohydrodynamic)	1–10 μm	~60–90%	Ultra-fine mesh networks, neural tissue scaffolds, nanopatterning	[123]
Inkjet (drop-on-demand)	20–100 μm	~80–95%	High-resolution cell patterns, thin tissue layers, drug screening models	[122]
Laser-assisted (LAB)	10–50 μm	≥90%	High-precision cell placement, microscale co-cultures, vascular graft patches	[124,99]

**Table 4:** Materials Used for Scaffolding.

Category	Examples of Materials	References
Natural Polymer	Collagen, dECM, Gelatin, GelMa, Alginate, Agarose, Chitosan, Hyaluronic Acid, Silk Fibroin	[89,134,135]
Synthetic Polymers	Polycaprolactone (PCL), Polylactic Acid (PLA), Polyglycolic Acid (PGA), Poly(lactic-co-glycolic acid) (PLGA), Polyethylene Glycol (PEG), Polyurethane (PU), Polyvinyl Alcohol (PVA), Pluronic F127	[68,73,77]
Ceramics	Calcium Phosphate, Bioglass, Hydroxyapatite, Tricalcium Phosphate, Calcium Silicate, β-Tricalcium Phosphate	[69,136]
Metal	Magnesium and Magnesium-alloys, Zinc and Zinc-alloys, Titanium and Titanium alloys, Stainless Steel	[137,138,139]

## Data Availability

No new data were created or analyzed in this study. Data sharing is not applicable to this article.
